# Regioselective Monobromination of Phenols with KBr and ZnAl–BrO_3_^−^–Layered Double Hydroxides

**DOI:** 10.3390/molecules25040914

**Published:** 2020-02-18

**Authors:** Ligeng Wang, Chun Feng, Yan Zhang, Jun Hu

**Affiliations:** College of Chemical Engineering, Zhejiang University of Technology, Hangzhou 310004, China; 2111701029@zjut.edu.cn (C.F.); 2111801310@zjut.edu.cn (Y.Z.)

**Keywords:** phenol, bromination, regioselecitvity

## Abstract

The regioselective mono-bromination of phenols has been successfully developed with KBr and ZnAl–BrO_3_^−^–layered double hydroxides (abbreviated as ZnAl–BrO_3_^−^–LDHs) as brominating reagents. The para site is much favorable and the ortho site takes the priority if para site is occupied. This reaction featured with excellent regioselectivity, cheap brominating reagents, mild reaction condition, high atom economy, broad substrate scope, and provided an efficient method to synthesize bromophenols.

## 1. Introduction

Bromophenols are versatile starting materials in a carbon–carbon bond coupling reaction [1,2,3,4,5]. They are also important constituents of naturally occurring compounds that possess a range of biological activities [6,7,8,9], such as antioxidant, antimicrobial and anticancer effects. Cheap and active molecular bromine is a traditional brominating reagent despite it being corrosive, having a low atom economy (up to 50% in substitution reaction) and low selectivity [10,11,12]. Safer N-bromosuccinimide based analogues are developed as preferable brominating reagents [13,14,15,16,17,18,19]. However, these brominating reagents employ Br_2_ in preparation and produce useless organic waste [20,21]. Oxidative bromination is another approach to brominated substrates [22,23,24,25,26]. This brominating reagent is generated in situ from bromide ion in the presence of oxidants [27]. Various oxidants such as H_2_O_2_, DMSO, O_2_, bromate, oxone, persulfate, etc., have been applied in oxidative bromination [28,29,30,31,32,33,34]. 

**Figure 1:**
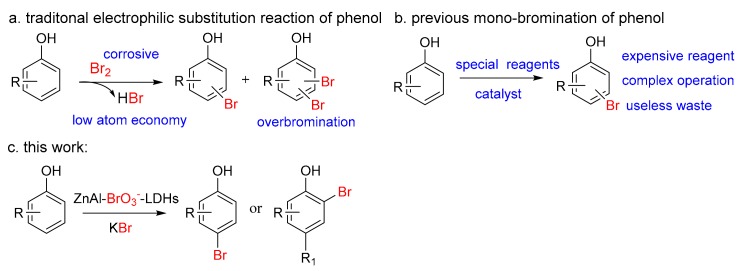
Three bromination of phenols; (**a**) traditional electrophilic substitution reaction of phenol; (**b**) previous mono-bromination of phenol; (**c**) this work.

Due to the high activity and multiple substituted sites of phenols, over-bromination frequently occurs when phenol derivatives undergo bromination [35,36]. It is usually hard to obtain mono-brominated product, as it is usually a mixture of mono- and multi-brominated substrate (Figure 1a). Numerous strategies for selective mono-bromination of phenols have been reported in literature.

Some methods use special brominating reagents or additives (Figure 1b), such as o-xylylene bis (triethyl ammonium tribromide) [35], bromotrimethylsilane and di-4-chlorophenyl-sulfoxide [37], (diacetoxyiodo)benzene and AlBr_3_ [38], *p*-toluenesulfonic acid [39,40,41], methanol [39,42], polyvinylpolypyrrolidone-Br_2_ [43], 1,3-di-n-butylimidazolium tribromide [44], ethylenebis(*N*-methylimidazolium) ditribromide [45], *N*-benzyl-triethylenediamine tribromide [46], 1-butyl-3-methylpyridiniumtribromide [47], ZrBr_4_/diazene [48], β-cyclodextrin [49] and tetrabromobenzene-1,3-disulfonylamide [50]. There are also some methods requiring a catalyst, including thioamide [36], Cu−Mn spinel oxide [51], bromoperoxidase [52], Rhenium-promoted mesoporous zirconia [53], amberlyst-15 [54], mesoporous silica supported sulfated zirconia [55], copper icons [56,57], UV–vis light irradiation [58] and ammonium acetate [59].

However, some of these methods are associated with disadvantages such as special and expensive reagents, demand for catalysts, generation of a large amount of waste, and harsh conditions. Therefore, the development of simple, efficient approaches to bromophenol is still highly desirable.

Layered double hydroxides (LDHs) is a layered structure material that consists of interlayer anions and positively charged layers [60]. LDHs have been applied in various fields [61,62,63,64,65]. In our early work, bromate has been successfully intercalated into LDHs and it is named ZnAl-BrO_3_^−^-layered double hydroxides (abbreviated as ZnAl–BrO_3_^−^–LDHs). 0.93 g ZnAl-BrO_3_^-^-LDHs is equivalent to 1 mmol bromate according to the indirect iodometric method [66]. Please see the specific synthetic procedure and calculation of iodometric method in the Supplementary Materials. We have utilized ZnAl–BrO_3_^−^–LDHs to achieve bromination of olefins and anilines [67,68,69]. Herein, we proposed a mild and efficient KBr and ZnAl–BrO_3_^−^–LDHs system for selective mono-bromination of phenols. 

## 2. Results

4-Methylphenol was chosen as the starting reactant and the optimization result is listed in Table 1. At first, the reaction was carried on at room temperature in presence of ZnAl–BrO_3_^–^–LDHs, lithium bromide and acetic acid. Maintaining the amount of ZnAl–BrO_3_^−^–LDHs at 0.2 equivalents, the amount of lithium bromide was changed from 1.4 to 0.8 equivalents. (Table 1, entry 1–4). The yield of mono-brominated product 1a altered from low to high then low and got the maximum 76% (entry 3). Then several different bromide salts (entry 5–7) were tested and potassium bromide gave the best yield in 83%. The brominated product was not observed in several common organic solvents which cannot provide acid environment (entry 8–11). This result demonstrates that acid is indispensable for our oxidative bromination system. Acetic acid plays a dual role as a solvent and acid provider. After adding a small amount of water, the reaction rate increased significantly and the yield slightly increased to 86% (entry 12). The possible reason is bromide salt and released bromate have good solubility in this mixed solvent. By screening reaction temperatures (entry 13–16), we determine 35 °C is sufficient to drive the reaction to completion and higher temperature cannot offer better yield. 

4-Methylphenol has two equal substituted sites, so double dosages of ZnAl–BrO_3_^−^–LDHs and potassium bromide were reasonable (entry 17). Surprisingly, the yield of desired di-brominated product 1b was only 17%, and 1a was still the main product 76% (entry 17). We conducted a control experiment that directly used potassium bromate (0.4 equiv.) and potassium bromide (2.0 equiv.) as brominating reagents. Not like the former result, 1a was not dominant and 1b was produced in large quantities in 61% (entry 18). Using potassium bromate (0.2 equiv.) and potassium bromide (1.0 equiv.) also produced 1b in 28% yield (entry 19). Without the addition of ZnAl-BrO_3_^−^-LDHs, brominated product was not obtained (entry 20). Our brominating system could make the bromination stop at monobromination stage by controlling the dosage of bromine. This is presumably due to the fact that open bromate produces large amounts of brominating reagents in a short period of time, while the special structure of LDHs can achieve slow release of BrO_3_^−^ under acetic acid conditions. This series of experiments demonstrate that our reagents can achieve mono-bromination in good yield.

## 3. Discussion

**Figure 2:**
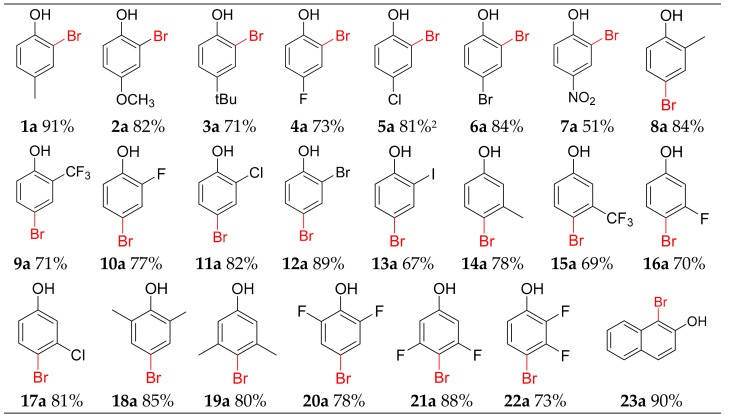
Scope of bromination.

To extend the substrate scope, some similar para-substituted phenols were chosen as substances (Figure 2). Our reagents show good tolerance for electron-withdrawing and electron-donating group. Phenol derivatives bearing methoxy and tert-butyl substituent were mono-brominated in ortho site to offer **2a** and **3a** in 82% and 71% yield. The bromination of phenol derivatives, which contain weak electronic withdrawing fluoro, chloro and bromo substituent at the para position, produced corresponding mono-brominated **4a**–**6a** in 73%, 81%, and 84% yields, respectively. The inert 4-nitrophenol provided **7a** in 51% yield.

Several ortho-substituted phenol derivatives performed well producing bromophenols. Phenol derivatives bearing methyl, trifluoromethyl were all mono-brominated to furnish selective para-brominated products **8a**–**9a** in 71%–84% yield. The halogen-containing derivatives could also be regioselectively brominated at para site to offer **10a**–**13a** (67–89%). These ortho-substituted phenol derivatives all have two different substituted sites, but para-substituted product was dominant, the and ortho- and multi-brominated products were not observed. This result confirmed our method could achieve mono-bromination with an excellent yield and high regioselectivity.

Starting from meta-substituted derivatives 3-methyl, 3-trifluoromethyl, 3-fluoro and 3-chlorophenol, 78%, 69%, 70% and 81% yield were obtained for **14a**–**17a**. The para site is still the main substituted position. Other para-open phenol derivatives 2,6-dimethylphenol, 3,5-dimethylphenol, 2,6-difluorophenol, 3,5-difluorophenol and 2,3-difluorophenol, were mono-brominated at the para site to offer **18a**–**22a** in high yield (78%–90%). The bromination of 2-naphthol afforded **23a** in a 90% yield.

**Figure 3:**
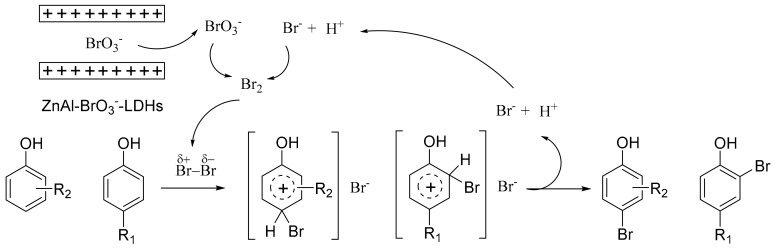
Proposed mechanism of bromination.

On the basis of the previous reports, the possible mechanism of monobromination of phenols is proposed in Figure 3. Bromate is stored in LDHs and bromide is open and abundant in solvent, thus the bromide is the excess in the redox reaction between them. Released bromate reacts with bromide to produce Br_2_. Polarization and heterolytic cleavage occurred immediately in solvent. Then, the electrophilic substitution reaction is executed. Bromide and hydrogen that detached from phenol are used for next oxidative cycle. ZnAl-BrO_3_**^−^**–LDHs achieve smooth and slow release of brominating reagent, avoiding a high brominating concentration and violent reaction in the beginning.

## 4. Materials and Methods 

### 4.1. General Information

All reagents and solvents were purchased from commercial suppliers and were not purified. The crude products were purified by column chromatography on silica gel (Aladdin Industrial Corporation, Shanghai, China). Melting points of the compounds were recorded on X-4 melting point detector (Beijing Tech Instrument Co. Ltd., Beijing, China) and are uncorrected. ^1^H NMR spectra were recorded on a Bruker 500 MHz (Beijing, China) and a 400 MHZ spectrometer (Beijing, China) in deuterated chlororoform using tetramethylsilane as the internal standard. ^13^C-NMR spectra (Beijing, China) were recorded at 126 MHz and 101 MHz spectrometer in deuterated chlororoform solutions. Chemical shifts (δ) and coupling constants (J) were expressed in ppm and Hz, respectively. The high-resolution mass analyses were recorded on an Agilent high resolution mass spectrometer (Shanghai, China).

### 4.2. General Procedure for the Bromination

0.11 g (1.0 mmol) of 4-methylphenol, 5 mL of acetic acid, 0.5 mL of water, and 0.12 g (1.0 mmol) potassium bromide were placed in round-bottomed flask. Then, 0.19 g (0.2 mmol) ZnAl–BrO_3_**^–^**–LDHs was added in the flask under stirring at 35 °C. After the addition, stirring was continued to the end of reaction (monitored by thin layer chromatography). The residual ZnAl–BrO_3_**^−^**–LDHs were removed by centrifugation. The product was extracted with 3 × 10 mL dichloromethane. The combined extract was washed with sodium sulfite solution, brine, and dried (Na_2_SO4). Evaporation of the solvent left the crude product. The crude product was purified by column chromatography over silica gel (ethyl acetate-petroleum ether) to obtain pure product.

## 5. Conclusions

In conclusion, we developed a selective and mild mono-bromination of phenols with ZnAl-BrO_3_**^−^**–LDHs and potassium bromide as brominating reagents. Our strategy selectively produced para-brominated phenols, unless the para-position is substituted. As for para-substituted phenols, ortho-brominated products are obtained. The mild condition, simple experiment operation, high bromide atom economy, cheap brominating reagents, as well as no catalyst, make the present strategy prospective for the mono-bromination of phenols. 

## Figures and Tables

Unless otherwise noted, the mixture of substrate (1 mmol), ZnAl-BrO_3_**^–^**–LDHs (0.2 mmol), KBr (1.0 mmol) in AcOH/H_2_O (5mL/0.5mL) was stirred at 35 °C. Isolated yield.^2^ The reaction temperature was 50 °C.

**Table 1:** Optimization of bromination.^1^

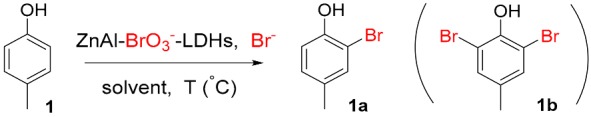					
Entry	Oxidant (equiv.)	Bromide (equiv.)	Solvent ^2^	Temperature	Yield ^3^
1	ZnAl-BrO_3_^-^-LDHs (0.2)	LiBr (1.4)	AcOH	25 °C	71
2	ZnAl-BrO_3_^-^-LDHs (0.2)	LiBr (1.2)	AcOH	25 °C	73
3	ZnAl-BrO_3_^-^-LDHs (0.2)	LiBr (1.0)	AcOH	25 °C	76
4	ZnAl-BrO_3_^-^-LDHs (0.2)	LiBr (0.8)	AcOH	25 °C	68
5	ZnAl-BrO_3_^-^-LDHs (0.2)	NaBr (1.0)	AcOH	25 °C	80
6	ZnAl-BrO_3_^-^-LDHs (0.2)	KBr (1.0)	AcOH	25 °C	83
7	ZnAl-BrO_3_^-^-LDHs (0.2)	ZnBr_2_ (0.5)	AcOH	25 °C	73
8	ZnAl-BrO_3_^-^-LDHs (0.2)	KBr (1.0)	MeOH	25 °C	-
9	ZnAl-BrO_3_^-^-LDHs (0.2)	KBr (1.0)	EtOH	25 °C	-
10	ZnAl-BrO_3_^-^-LDHs (0.2)	KBr (1.0)	DCM	25 °C	-
11	ZnAl-BrO_3_^-^-LDHs (0.2)	KBr (1.0)	EA	25 °C	-
12	ZnAl-BrO_3_^-^-LDHs (0.2)	KBr (1.0)	AcOH/H_2_O	25 °C	86
13	ZnAl-BrO_3_^-^-LDHs (0.2)	KBr (1.0)	AcOH/H_2_O	30 °C	85
14	ZnAl-BrO_3_^-^-LDHs (0.2)	KBr (1.0)	AcOH/H_2_O	35 °C	91
15	ZnAl-BrO_3_^-^-LDHs (0.2)	KBr (1.0)	AcOH/H_2_O	40 °C	81
16	ZnAl-BrO_3_^-^-LDHs (0.2)	KBr (1.0)	AcOH/H_2_O	45 °C	77
17	ZnAl-BrO_3_^-^-LDHs (0.4)	KBr (2.0)	AcOH/H_2_O	35°C	76 (17)
18	KBrO_3_ (0.4)	KBr (2.0)	AcOH/H_2_O	35 °C	22 (61)
19	KBrO_3_ (0.2)	KBr (1.0)	AcOH/H_2_O	35 °C	35 (28)
20	-	KBr (1.0)	AcOH/H_2_O	35 °C	-

^1^ Phenol bromination condition was as following: 4-methylphenol (1mmol), ZnAl-BrO_3_**^–^**–LDHs, bromide ion, AcOH (5mL). ^2^ 0.5 mL of water was added in entries 13–20. ^3^ Isolate yield of **1a**. Number in parentheses is the yield of **1b**.

## Supplementary Materials

The following are available online at https://www.mdpi.com/1420-3049/25/4/914/s1, general procedure for ZnAl-BrO_3_^-^-LDHs, characterization data, ^1^H NMR and ^13^C NMR Spectra of products.
