# Supplementary material for: Regioselective Monobromination of Phenols with KBr and ZnAl–BrO3−–Layered Double Hydroxides
Source: Molecules. 2020 Feb 18;25(4):914. doi: 10.3390/molecules25040914 (PMC7070364; doi:10.3390/molecules25040914)

Supplementary Material

Regioselective Monobromination of Phenols with ZnAl-BrO_3^-–LDHs and KBr

Ligeng Wang [1,*], Chun Feng [1], Yan Zhang [1] and Jun Hu[1,*]

[1] College of Chemical Engineering, Zhejiang University of Technology, Hangzhou, 310004, China

* Correspondence: wanglg@zjut.edu.cn ; hjzjut@zjut.edu.cn ;

Table of Contents **Page No.**

Table of Contents	Page No.
**General Procedure for ZnAl-BrO_3^--LDHs and iodometric method**	**2**
**Characterization data for products**	**2-5**
**^{1}H NMR and ^{13}C NMR Spectra of products**	**6-29**

General Procedure for ZnAl-BrO_3^--LDHs

Bromate intercalated ZnAl-layered double hydroxides (ZnAl-BrO_3^--LDHs) were synthesized by the coprecipitation method according to the literature (Journal of the Chinese Ceramic Society, 2015, 43, 672-677). The procedure need a 500 mL four-necks-flask, a mechanical stirrer and two dropping funnels. 8.35 g $KBrO_3$ (AR, 0.05 mol) was dissolved in 100 mL water, then the solution was added to the four-necks-flask. $Al(NO_3)_3{\cdot}6H_2O$ (18.75 g, 0.05 mol) and $Zn(NO_3)_2{\cdot}9H_2O$ (29.75 g, 0.10 mol) were dissolved in 200 mL water (Solution A). NaOH (12.00 g ,0.30 mol) were dissolved in 200 mL water (Solution B). Solution A and B were moved to two dropping funnels, then were added to the four-necks-flask with temperature at 30°C. During the addition progress, keep pH at 7± 0.2 by adjust drop rate of two solutions. After the addition, the mixture was stirred constantly at 30°C for 1 h and then crystallized at 70°C for 24 h. The crude product was filtered and washed with deionized water repeatedly until the solution pH at 7. The wet was dried at 60°C for 18 h, and the product was recorded as ZnAl-BrO_3^--LDHs.

Iodometric method

Weigh a certain mass of ZnAl-BrO_3^--LDHs. Use excess sulfuric acid to release all bromate. Released bromate and KI undergo redox reaction to form I_2 and bromide. Calibrate I_2 with standard $Na_2S_2O_3$ solution. Calculate the result according to the reaction formula below. The final result is 0.93g ZnAl-BrO_3^--LDHs is equivalent to 1mmol BrO_3^-.

$$2\ BrO_3^- + 10\ I^- + 12H^+ = 5I_2 + 2\ Br^- + 6H_2O$$

$$I_2 + 2\ S_2O_3^{2-} = 2I^- + S_4O_6^{2-}$$

$$c(BrO_3^-) = \frac{c(Na_2S_2O_3) \times V(Na_2S_2O_3)}{5 \times m(LDHs)}$$

Characterization Data of Products (1a-23a, 1b)

2-bromo-4-methylphenol (1a): 0.17g (91%), off-white needle crystalline; mp 55-57°C; ^{1}H NMR (500 MHz, $CDCl_3$) δ 7.29 (d, *J* = 1.4 Hz, 1H, H-2), 7.10 – 6.97 (m, 1H, H-4), 6.94 (s, 1H, H-6), 5.42 (s, 1H, OH), 2.29 (s, 3H, CH_3); ^{13}C NMR (126 MHz, $CDCl_3$) δ 149.97(C, C-1), 131.75(C, C-3), 131.39(C, C-4), 129.73(C, C-5), 115.74(C, C-6), 109.79(C, C-2), 20.14(C, CH_3); HREIMS m/z 186.9756 (calcd for C_7H_7Br, 186.9758).

2,6-dibromo-4-methylphenol (1b): white crystalline powder; mp 49-50°C; ^{1}H NMR (500 Hz, $CDCl_3$) δ 7.41 – 7.15 (m, 2H, H-3,5), 5.74 (s, 1H, OH), 2.27 (s, 3H, CH_3); ^{13}C NMR (126 MHz, $CDCl_3$) δ 147.09(C, C-1), 132.33(C, C-3,4,5), 109.40(C, C-2,6), 19.93(C, CH_3); HREIMS m/z 264.8864 (calcd for $C_7H_6Br_2$, 264.8863).

2-bromo-4-methoxyphenol (2a): 0.17g (82%), off-white crystalline: mp 46-48°C; ^{1}H NMR (500 MHz, $CDCl_3$) δ 7.03 (d, *J* = 2.9 Hz, 1H, H-3), 6.96 (d, *J* = 8.9 Hz, 1H, H-5), 6.81 (dd, *J* = 8.9, 2.9 Hz, 1H, H-6), 3.77 (s, 3H, OCH_3); ^{13}C NMR (126 MHz, $CDCl_3$) δ 153.75(C, C-4), 146.47(C, C-1), 116.85(C, C-3), 116.33(C, C-6), 115.28, 1(C,

C-5)09.89(C, C-2), 55.95(C, OCH_3); HREIMS m/z 202.9706 (calcd for C_7H_8OBr, 202.9707).

2-bromo-4-(tert-butyl) phenol (3a): 0.16g (71%), colorless liquid; ^{1}H NMR (500 MHz, $CDCl_3$) δ 7.50 (d, *J* = 2.3 Hz, 1H, H-5), 7.28 (dd, *J* = 8.5, 2.3 Hz, 1H, H-3), 7.01 (d, *J* = 8.5 Hz, 1H, H-6), 5.57 (s, 1H, OH), 1.33 (s, 9H, t-Bu); ^{13}C NMR (126 MHz, $CDCl_3$) δ 149.82(C, C-1), 145.00(C, C-4), 128.77(C, C-3), 126.13(C, C-5), 115.59(C, C-6), 109.82(C, C-2), 34.10(C, $C(CH_3)_3$), 31.33(C, $C(CH_3)_3$); HREIMS m/z 229.0225 (calcd for $C_{10}H_{13}Br$, 229.0228).

2-bromo-4-fluorophenol (4a): 0.14g (73%),light brown powder: mp 42-43°C; ^{1}H NMR (500 MHz, $CDCl_3$) δ 7.23 (ddd, *J* = 7.7, 2.5, 0.5 Hz, 1H, H-3), 7.06 – 6.87 (m, 2H, H-5,6), 5.41 (s, 1H, OH); ^{13}C NMR (126 MHz, $CDCl_3$) δ 156.36(C, C-4), 155.39(C, C-1), 148.83(C, C-3), 118.66(C, C-6), 116.13(C, C-5), 109.51(C, C-2); HREIMS m/z 190.9507 (calcd for C_6H_4BrF, 190.9508).

2-bromo-4-chlorophenol (5a): 0.17g (81%), white crystalline; mp 30-33°C; ^{1}H NMR (500 MHz, $CDCl_3$) δ 7.48 (d, *J* = 2.5 Hz, 1H, H-3), 7.21 (dd, *J* = 8.7, 2.5 Hz, 1H, H-5), 6.97 (d, *J* = 8.7 Hz, 1H), H-6, 5.54 (s, 1H, OH); ^{13}C NMR (126 MHz, $CDCl_3$) δ 151.16(C, C-1), 131.36(C, C-3), 129.21(C, C-5), 125.8(C, C-4), 116.89(C, C-6), 110.35(C, C-2); HREIMS m/z 206.9210 (calcd for C_6H_4BrCl, 206.9212).

2,4-dibromophenol (6a): 0.21g (84%), white crystalline; mp 37-39°C; ^{1}H NMR (400 MHz, Chloroform-*d*) δ 7.57 (d, *J* = 2.4 Hz, 1H, H-3), 7.30 (dd, *J* = 8.7, 2.4 Hz, 1H, H-5), 6.88 (d, *J* = 8.7 Hz, 1H, H-6), 5.56 (d, *J* = 2.4 Hz, 1H, OH); ^{13}C NMR (101 MHz, Chloroform-*d*) δ 151.61(C, C-1), 134.08(C, C-3), 132.14(C, C-5), 117.49(C, C-6), 112.73(C, C-4), 110.90(C, C-2); HREIMS m/z 250.8709 (calcd for $C_6H_4Br_2$, 250.8707).

2-bromo-4-nitrophenol (7a): 0.11g (51%), light yellow crystalline; mp 111-114°C; ^{1}H NMR (500 MHz, $CDCl_3$) δ 8.45 (d, *J* = 2.6 Hz, 1H, H-3), 8.17 (dd, *J* = 9.0, 2.6 Hz, 1H, H-5), 7.14 (d, *J* = 9.0 Hz, 1H, H-6), 6.36 (s, 1H, OH); ^{13}C NMR (126 MHz, $CDCl_3$) δ 157.92(C, C-1), 141.75(C, C-4), 128.40(C, C-3), 125.30(C, C-5), 114.25(C, C-6), 110.06(C, C-2); HREIMS m/z 217.9451 (calcd for $C_6H_4BrNO_2$, 217.9453).

4-bromo-2-methylphenol (8a): 0.16g (84%), white crystalline; mp 64-66°C; ^{1}H NMR (500 MHz, CDCl3) δ 7.31 – 7.22 (m, 1H, H-3), 7.18 (dd, J = 8.5, 2.4 Hz, 1H, H-5), 6.66 (d, J = 8.5 Hz, 1H, H-6), 5.10 (s, 1H, OH), 2.23 (s, 3H); ^{13}C NMR (126 MHz, CDCl3) δ 152.85(C, C-1), 133.52(C, C-3), 129.69(C, C-2), 126.36(C, C-6), 116.56(C, C-5), 112.55(C, C-4), 15.61(C, CH_3); HREIMS m/z 186.9756 (calcd for C_7H_7Br, 186.9758).

4-bromo-2-(trifluoromethyl) phenol (9a): 0.17g (71%), white crystalline; mp 83-85°C; ^{1}H NMR (400 MHz, Chloroform-*d*) δ 7.63 (d, *J* = 2.4 Hz, 1H, H-3), 7.51 (dd, *J* = 8.8, 2.4 Hz, 1H, H-5), 6.84 (d, *J* = 8.7 Hz, 1H, H-6), 5.55 (d, *J* = 2.0 Hz, 1H, OH); ^{13}C NMR (101

MHz, Chloroform-*d*) δ 152.59(C, C-1), 136.43(C, C-3), 129.68(C, C-5), 123.20(C, C-6), 119.66(C, C-2), 118.27(C, CF_3), 112.73(C, C-4); HREIMS m/z 240.9474 (calcd for $C_7H_4BrF_3$, 240.9476).

4-bromo-2-fluorophenol (10a): 0.15g (77%), colorless liquid; ^{1}H NMR (500 MHz, $CDCl_3$) δ 7.25 – 7.20 (m, 1H, H-3), 7.02 – 6.92 (m, 2H, H-5,6), 5.38 (d, J = 16.4 Hz, 1H, OH); ^{13}C NMR (126 MHz, $CDCl_3$) δ 157.34(C, C-2), 155.42(C, C-1), 148.88(C, C-5), 118.79(C, C-3), 116.39(C, C-6), 109.57(C, C-4); HREIMS m/z 190.9510 (calcd for C_6H_4BrF, 190.9508).

4-bromo-2-chlorophenol (11a): 0.17g (82%), off-white crystalline; mp 47-49°C; ^{1}H NMR (500 MHz, $CDCl_3$) δ 7.47 (d, J = 2.3 Hz, 1H, H-3), 7.33 – 7.21 (m, 1H, H-5), 6.92 (d, J = 8.7 Hz, 1H, H-6), 5.62 (s, 1H, OH); ^{13}C NMR (126 MHz, $CDCl_3$) δ 150.70(C, C-1), 131.38(C, C-3,5), 120.85(C, C-2), 117.65(C, C-6), 112.30(C, C-4); HREIMS m/z 206.9214 (calcd for C_6H_4BrCl, 206.9212).

2,4-dibromophenol (12a): 0.22g (89%), white crystalline; mp 37-39°C; ^{1}H NMR (400 MHz, Chloroform-*d*) δ 7.57 (d, J = 2.4 Hz, 1H, H-3), 7.30 (dd, J = 8.7, 2.4 Hz, 1H, H-5), 6.88 (d, J = 8.7 Hz, 1H, H-6), 5.56 (d, J = 2.4 Hz, 1H, OH); ^{13}C NMR (101 MHz, Chloroform-*d*) δ 151.61(C, C-1), 134.08(C, C-3), 132.14(C, C-5), 117.49(C, C-6), 112.73(C, C-4), 110.90(C, C-2); HREIMS m/z 250.8708 (calcd for $C_6H_4Br_2$, 250.8707).

4-bromo-2-iodophenol (13a): 0.20g (67%), white crystalline; mp 88-90°C; ^{1}H NMR (500 MHz, $CDCl_3$) δ 7.78 (d, J = 2.3 Hz, 1H, H-3), 7.36 (dd, J = 8.7, 2.3 Hz, 1H, H-5), 6.89 (d, J = 8.7 Hz, 1H, H-6), 5.41 (s, 1H, OH); ^{13}C NMR (126 MHz, $CDCl_3$) δ 154.20(C, C-1), 139.78(C, C-3), 133.06(C, C-5), 116.30(C, C-6), 113.06(C, C-4), 86.12(C, C-2); HREIMS m/z 298.8567 (calcd for C_6H_4BrI, 298.8568).

4-bromo-3-methylphenol (14a): 0.15g (78%), off-white crystalline; mp 59-61°C; ^{1}H NMR (500 MHz, $CDCl_3$) δ 7.36 (d, J = 8.6 Hz, 1H, H-5), 6.75 (d, J = 2.9 Hz, 1H, H-2), 6.57 (dd, J = 8.5, 3.0 Hz, 1H, H-6), 2.34 (s, 3H, CH_3); ^{13}C NMR (126 MHz, $CDCl_3$) δ 154.38(C, C-1), 139.21(C, C-3), 133.08(C, C-5), 117.82(C, C-4), 115.57(C, C-2), 114.51(C, C-6), 22.91(C, CH_3); HREIMS m/z 186.9759 (calcd for C_7H_7Br, 186.9758).

4-bromo-3-(trifluoromethyl) phenol (15a): 0.17g (69%), yellow crystalline; mp 44-46°C; ^{1}H NMR (500 MHz, $CDCl_3$) δ 7.54 (d, J = 8.7 Hz, 1H, H-5), 7.19 (d, J = 3.0 Hz, 1H, H-2), 6.88 (dd, J = 8.6, 2.9 Hz, 1H, H-6); ^{13}C NMR (126 MHz, $CDCl_3$) δ 154.68(C, C-1), 136.05(C, C-3), 131.24(C, C-5), 123.67(C, CF_3), 120.06(C, C-6), 115.38(C, C-4), 110.15(C, C-2); HREIMS m/z 240.9477 (calcd for $C_7H_4BrF_3$, 240.9476).

4-bromo-3-fluorophenol (16a): 0.13g (70%), pale yellow crystalline; mp 70-72°C; ^{1}H NMR (500 MHz, $CDCl_3$) δ 7.38 (dd, J = 8.6, 8.0 Hz, 1H, H-5), 6.68 (dd, J = 9.7, 2.8 Hz, 1H, H-2), 6.56 (ddd, J = 8.7, 2.8, 1.0 Hz, 1H, H-6), 5.37 (s, 1H, OH); ^{13}C NMR (126

MHz, $CDCl_3$) δ 160.42(C, C-3), 158.46(C, C-1), 133.61(C, C-5), 112.70(C, C-6), 104.56(C, C-2), 99.53(C, C-4); HREIMS m/z 190.9507 (calcd for C_6H_4BrF, 190.9508).

4-bromo-3-chlorophenol (17a): 0.17g (81%), off-white crystalline; mp 64-66°C; ^{1}H NMR (400 MHz, Chloroform-*d*) δ 7.43 (d, *J* = 8.7 Hz, 1H, H-5), 6.96 (d, *J* = 2.9 Hz, 1H, H-2), 6.63 (dd, *J* = 8.7, 2.9 Hz, 1H, H-6), 5.27 (s, 1H, OH); ^{13}C NMR (101 MHz, Chloroform-*d*) δ 155.13(C, C-1), 134.92(C, C-5), 134.14(C, C-3), 117.57(C, C-2), 115.63(C, C-6), 113.21(C, C-4); HREIMS m/z 206.9214 (calcd for C_6H_4BrCl, 206.9212).

4-bromo-2,6-dimethylphenol (18a): 0.17g (85%), light brown crystalline; mp 74-78°C; ^{1}H NMR (500 MHz, $CDCl_3$) δ 7.11 (s, 2H, H-3,5), 2.23 (s, 6H, H-CH_3); ^{13}C NMR (126 MHz, $CDCl_3$) δ 151.26(C, C-1), 130.95(C, C-3,5), 125.24(C, C-2,6), 111.99(C, C-4), 15.70(C, CH_3); HREIMS m/z 200.9913 (calcd for C_8H_9Br, 200.9915).

4-bromo-3,5-dimethylphenol (19a): 0.16g (80%), pink crystalline; mp 113-115°C; ^{1}H NMR (400 MHz, Chloroform-*d*) δ 6.57 (s, 2H, H-2,6), 5.11 (d, *J* = 3.3 Hz, 1H, OH), 2.33 (s, 6H, CH_3); ^{13}C NMR (101 MHz, Chloroform-*d*) δ 153.79(C, C-1), 139.53(C, C-3,5), 118.40(C, C-4), 115.28(C, C-2,6), 23.87(C, CH_3); HREIMS m/z 200.9914 (calcd for C_8H_9Br, 200.9915).

4-bromo-2,6-difluorophenol (20a): 0.16g (78%), white crystalline; mp 50-52°C; ^{1}H NMR (400 MHz, Chloroform-*d*) δ 7.10 – 7.00 (m, 2H, H-3,5); ^{13}C NMR (101 MHz, Chloroform-*d*) δ 151.95(C, C-2,6), 132.63(C, C-1), 115.70(C, C-4), 110.05(C, C-3,5); HREIMS m/z 208.9416 (calcd for $C_6H_3BrF_2$, 208.9413).

4-bromo-3,5-difluorophenol (21a): 0.18g (88%), gray crystalline; mp 72-76°C; ^{1}H NMR (500 MHz, $CDCl_3$) δ 6.60 – 6.40 (m, 2H, H-2,6), 6.07 (s, 1H, OH); ^{13}C NMR (126 MHz, $CDCl_3$) δ 161.25(C, C-3,5), 159.28(C, C-1), 100.36(C, C-2,6), 88.59(C, C-4); HREIMS m/z 208.9412 (calcd for $C_6H_3BrF_2$, 208.9413).

4-bromo-2,3-difluorophenol (22a): 0.15g (73%), white powder; mp 53-57°C; ^{1}H NMR (400 MHz, Chloroform-*d*) δ 7.17 (ddd, *J* = 9.3, 6.9, 2.4 Hz, 1H, H-5), 6.72 (ddd, *J* = 8.9, 8.0, 2.2 Hz, 1H, H-6), 5.48 (s, 1H, OH); ^{13}C NMR (101 MHz, Chloroform-*d*) δ 148.24, 144.32, 140.68, 127.19, 113.20, 100.40; HREIMS m/z 208.9414 (calcd for $C_6H_3BrF_2$, 208.9413).

1-bromonaphthalen-2-ol (23a): 0.20g (90%), off-white powder; mp 78-81°C; ^{1}H NMR (500 MHz, $CDCl_3$) δ 8.06 (d, *J* = 8.5 Hz, 1H, H-8), 7.76 (d, *J* = 8.8 Hz, 2H, H-3,5), 7.59 (ddd, *J* = 8.3, 7.0, 1.1 Hz, 1H, H-6), 7.44 – 7.38 (m, 1H, H-7), 7.31 – 7.27 (m, 1H, H-2), 5.98 (s, 1H, OH). ^{13}C NMR (126 MHz, $CDCl_3$) δ 150.57(C, C-1), 132.29(C, C-9), 129.68(C, C-3,4), 127.81(C, C-2), 125.31(C, C-5,7), 124.12(C, C-8), 117.15(C, C-6), 106.13(C, C-10); HREIMS m/z 222.9755 (calcd for $C_{10}H_7Br$, 222.9758).

^{1}H NMR and ^{13}C NMR Spectra of products

1a 2-bromo-4-methylphenol

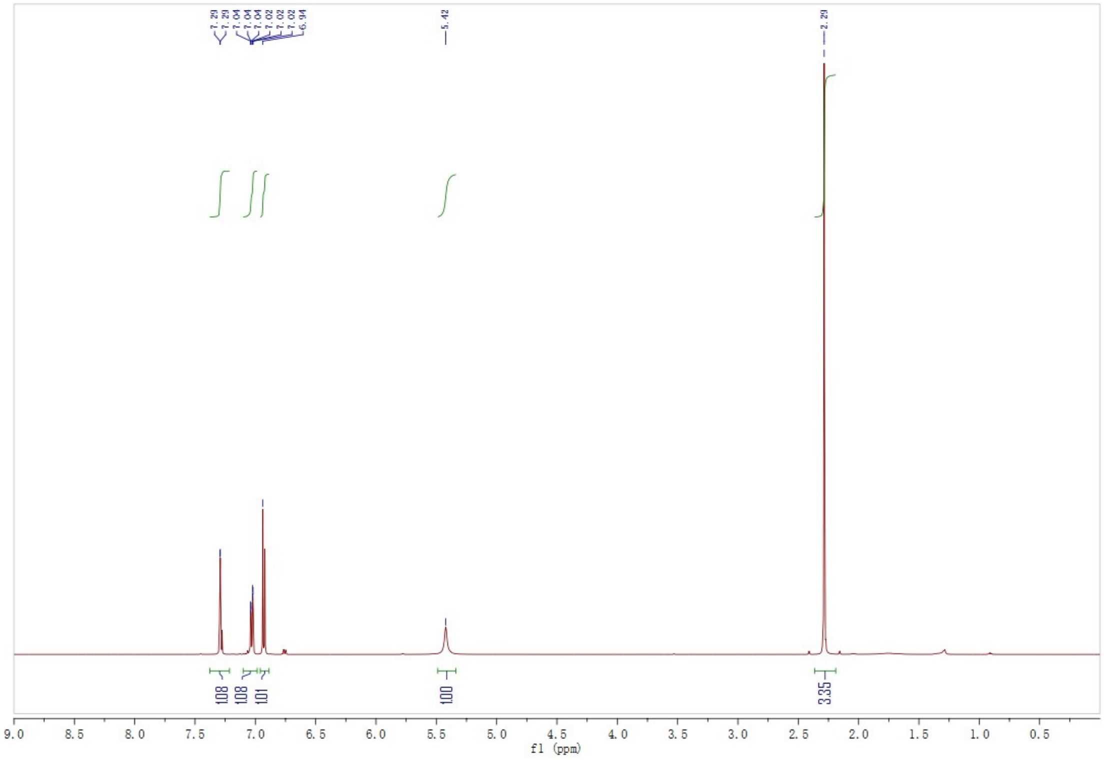

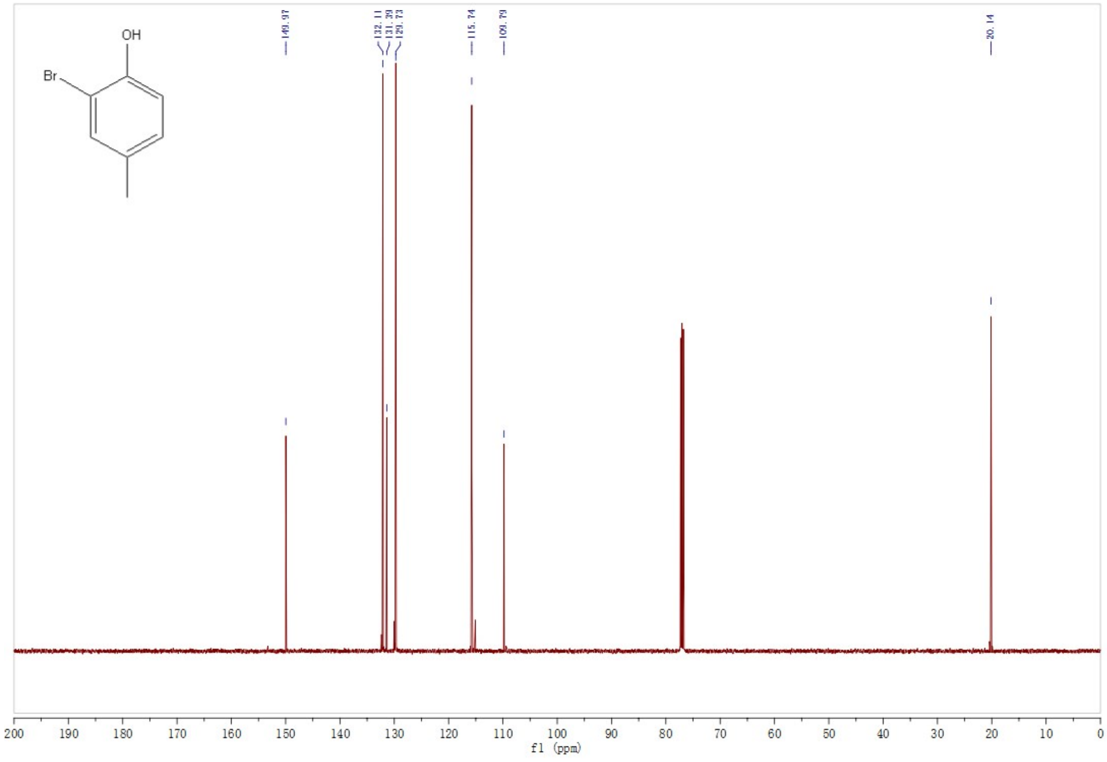

1b 2,6-dibromo-4-methylphenol

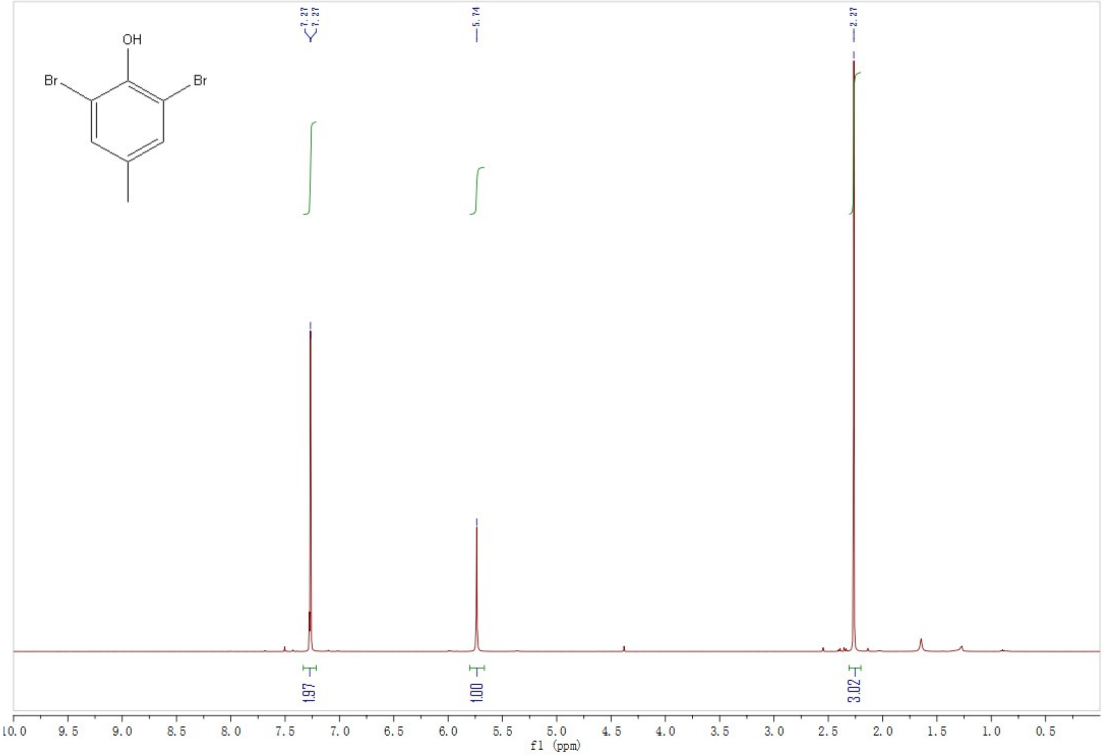

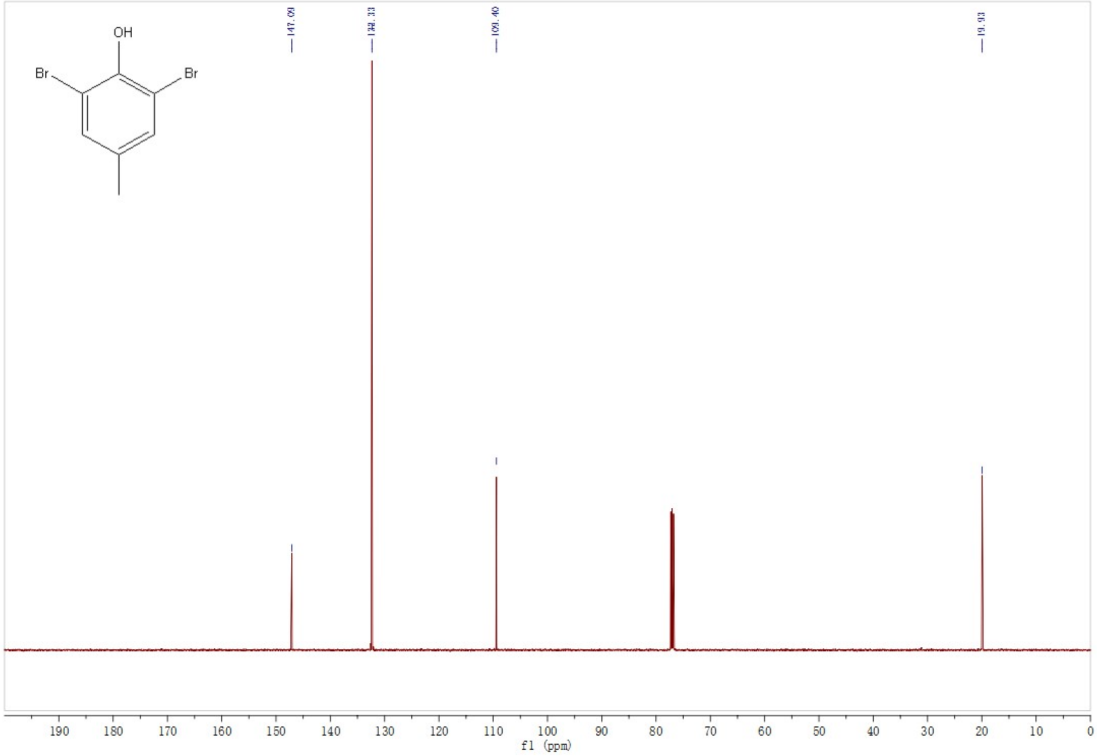

2a 2-bromo-4-methoxyphenol

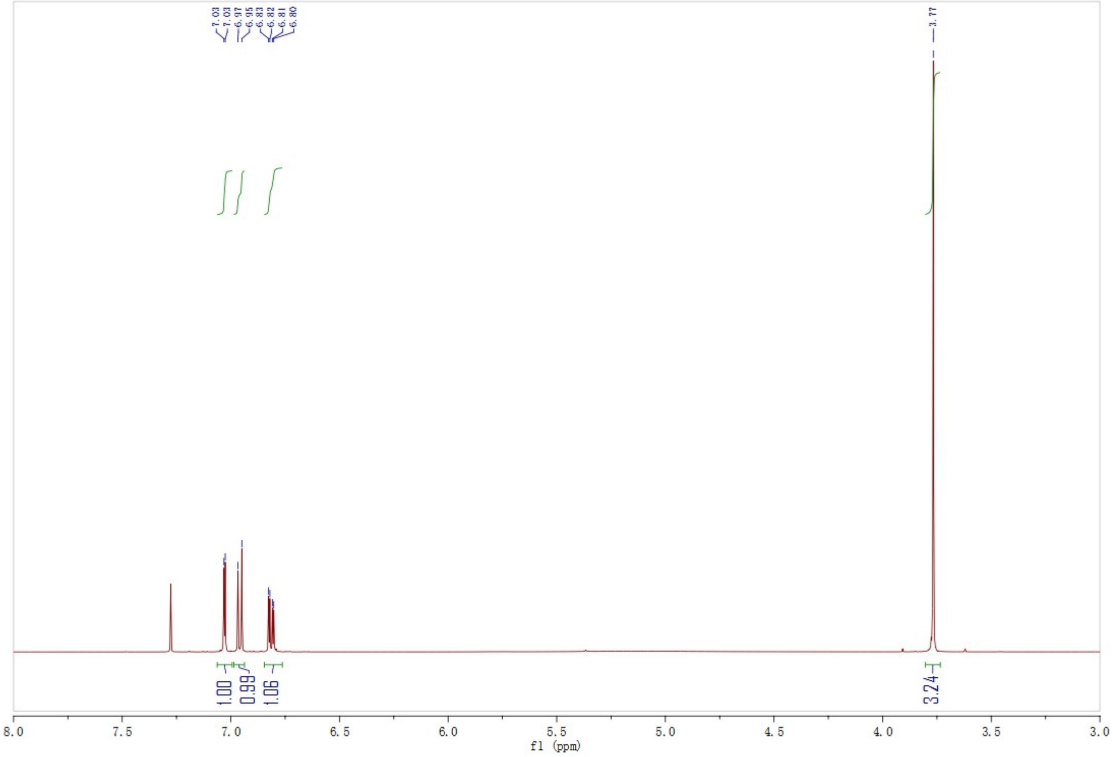

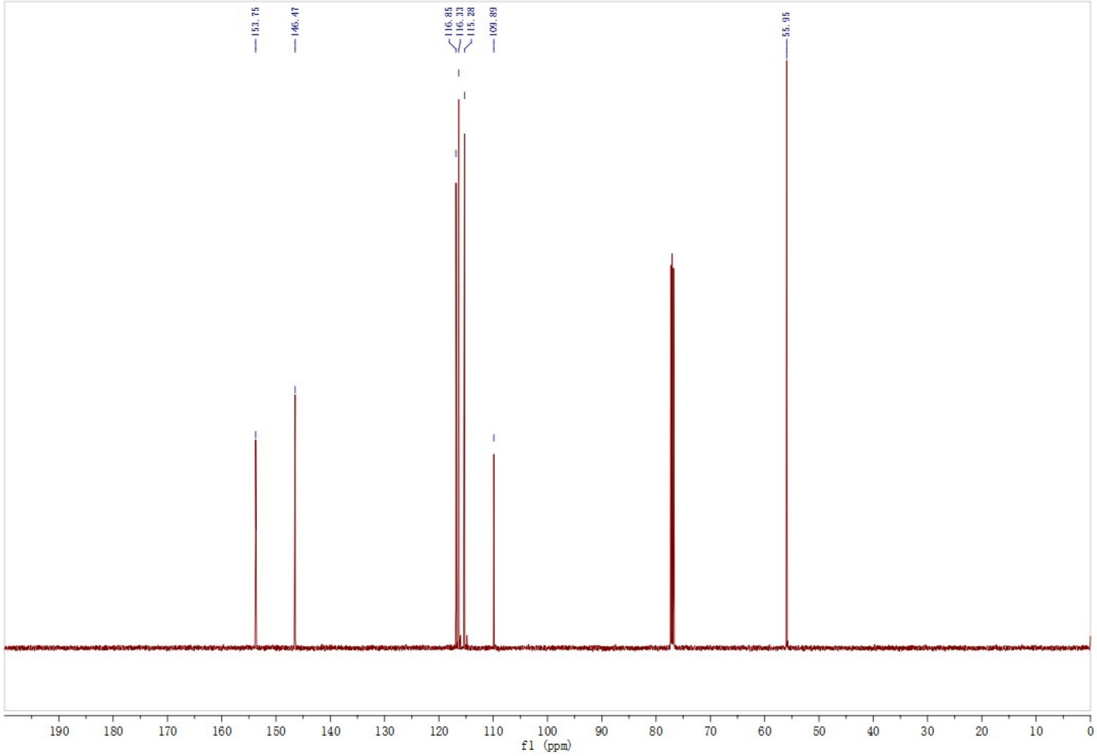

3a 2-bromo-4-(tert-butyl)phenol

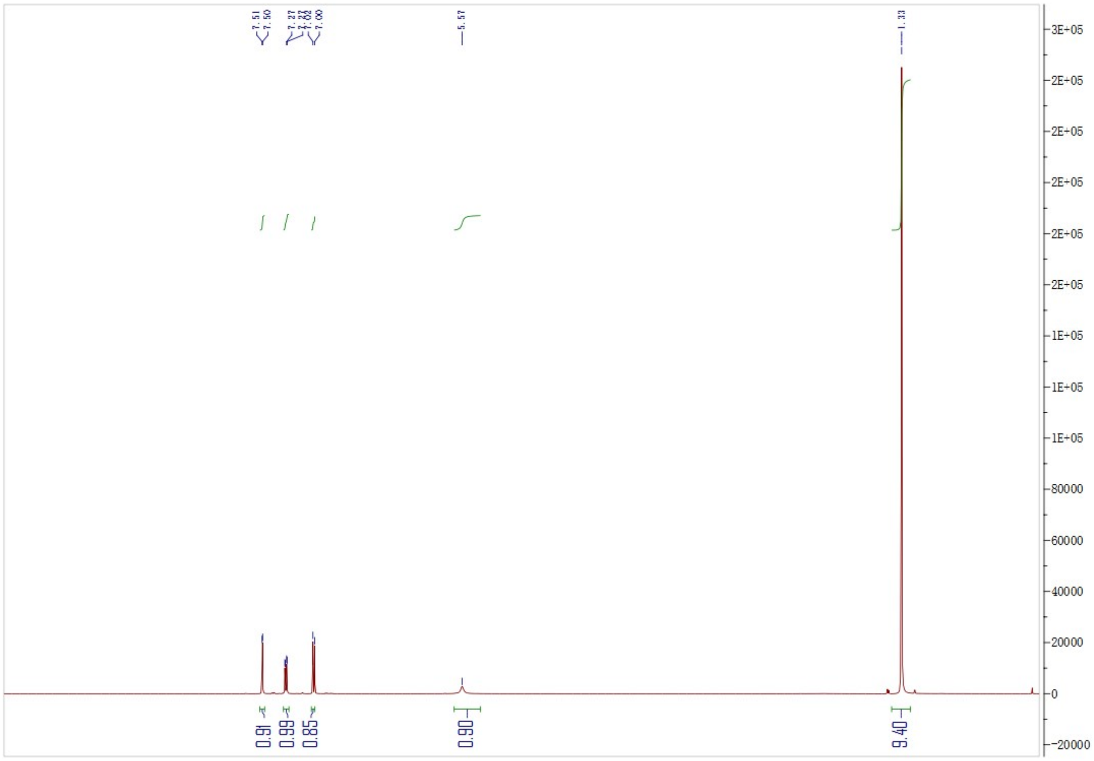

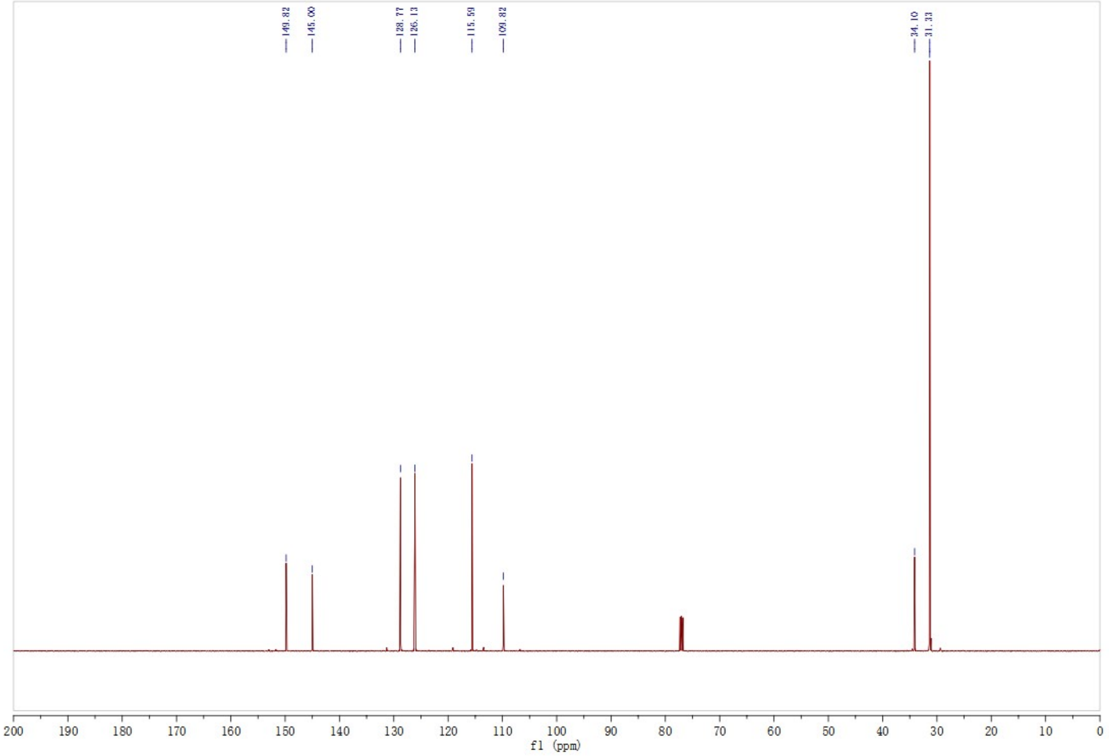

4a 2-bromo-4-fluorophenol

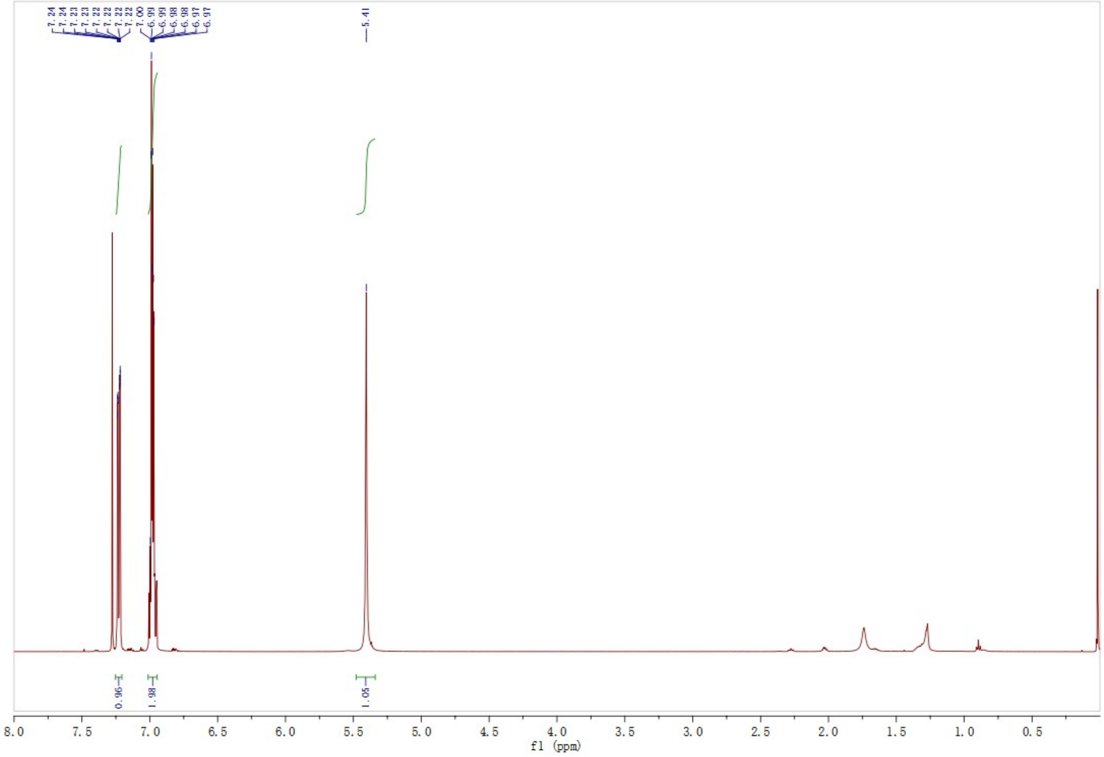

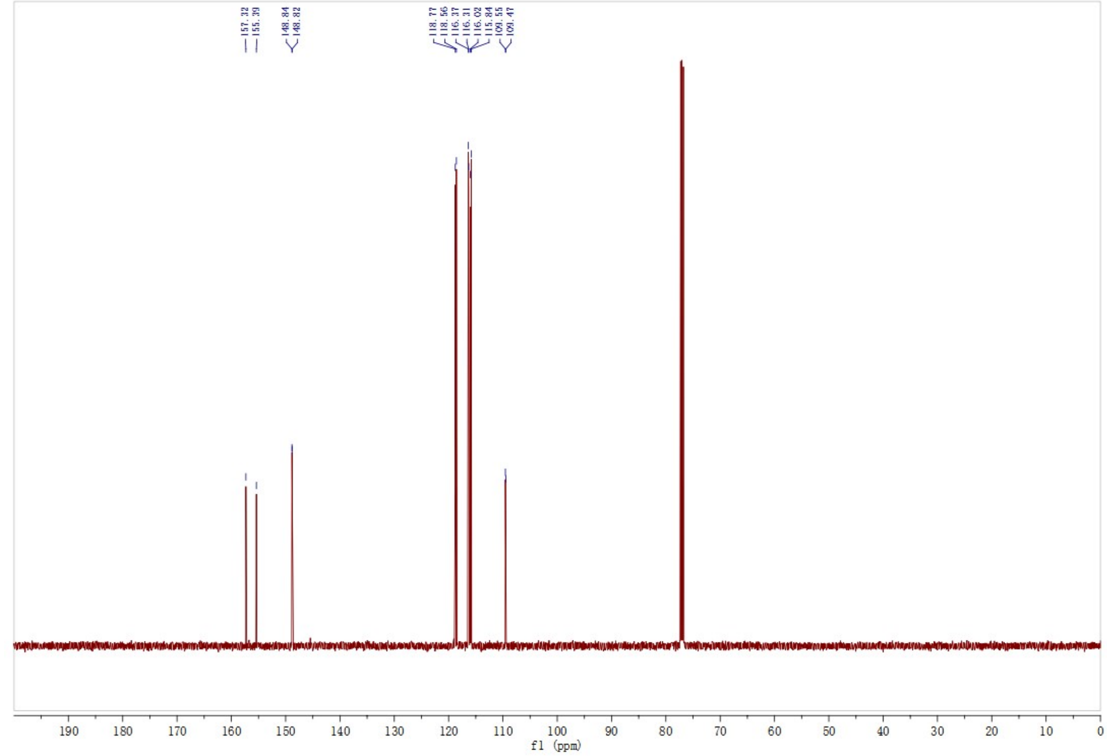

5a 2-bromo-4-chlorophenol

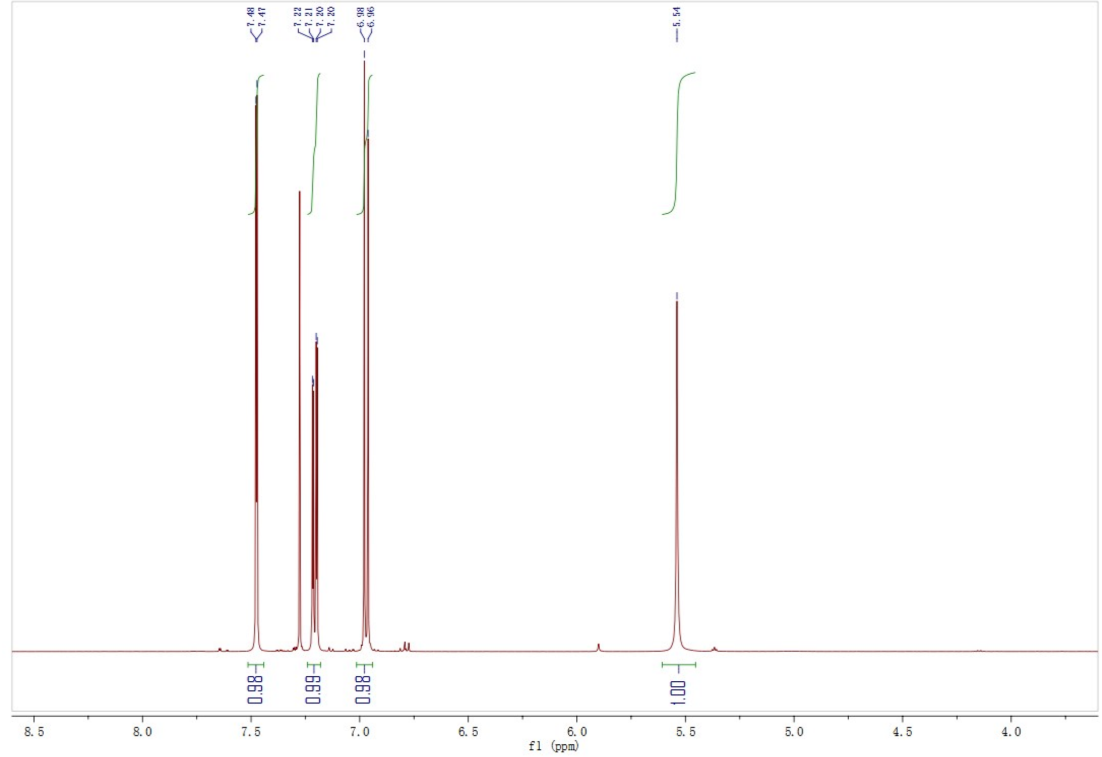

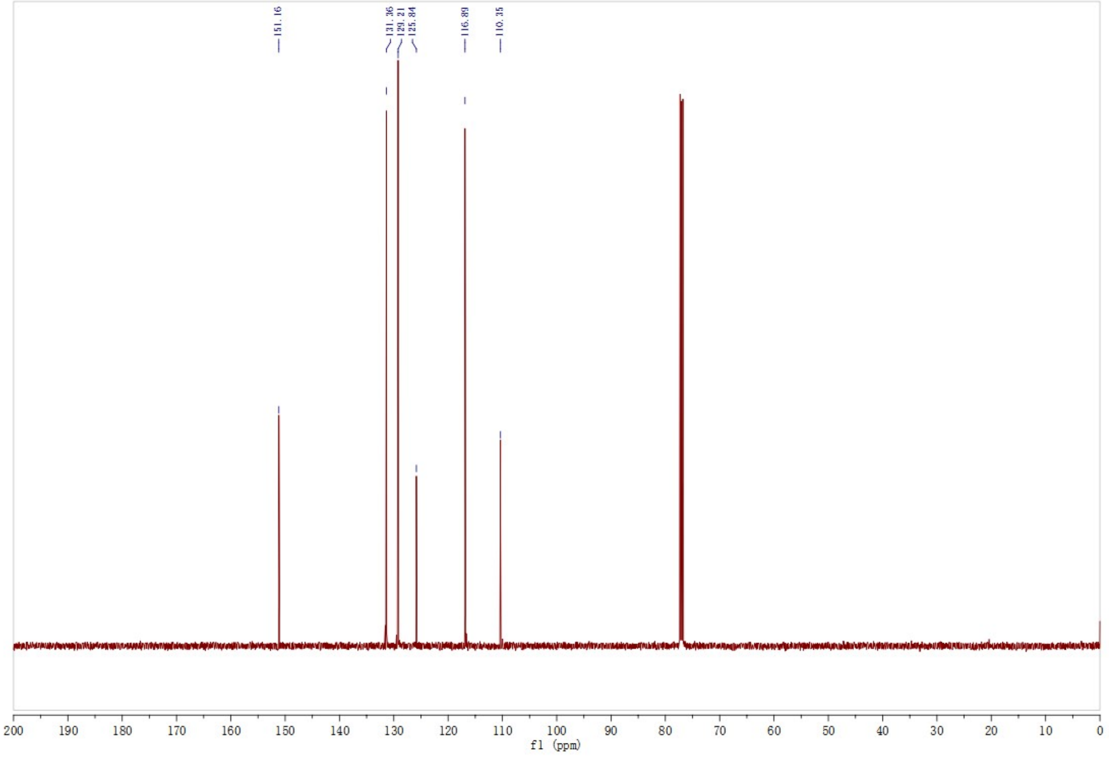

6a 2,4-dibromophenol

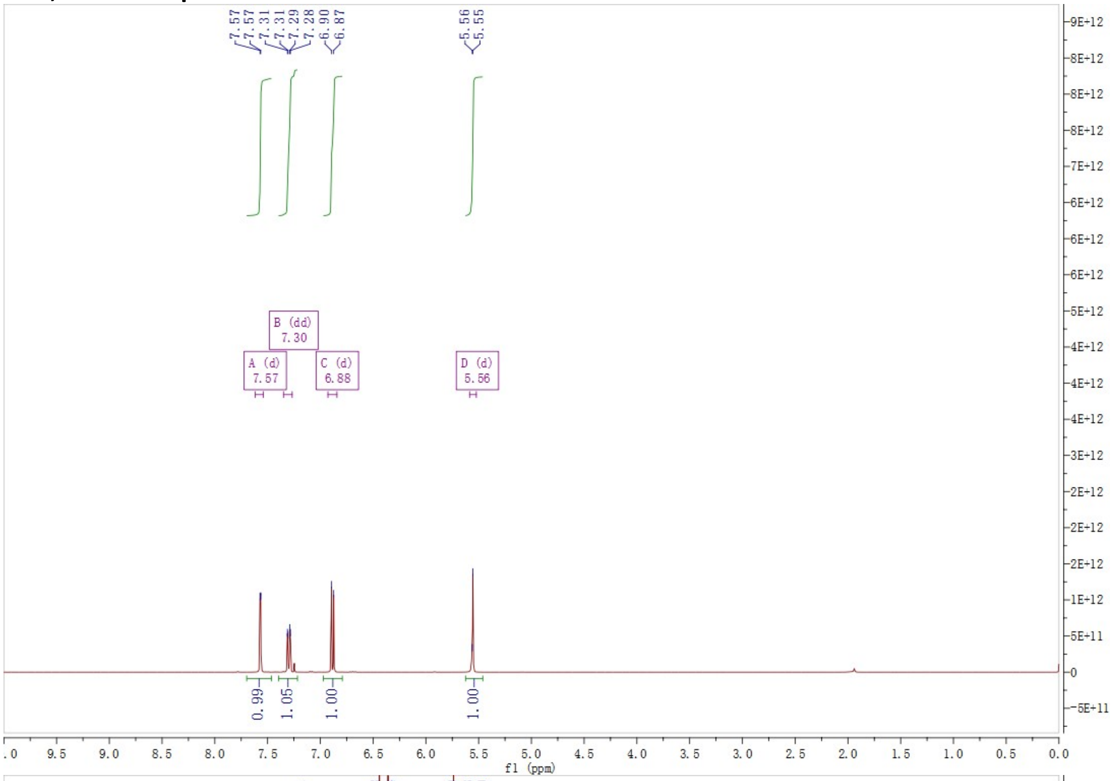

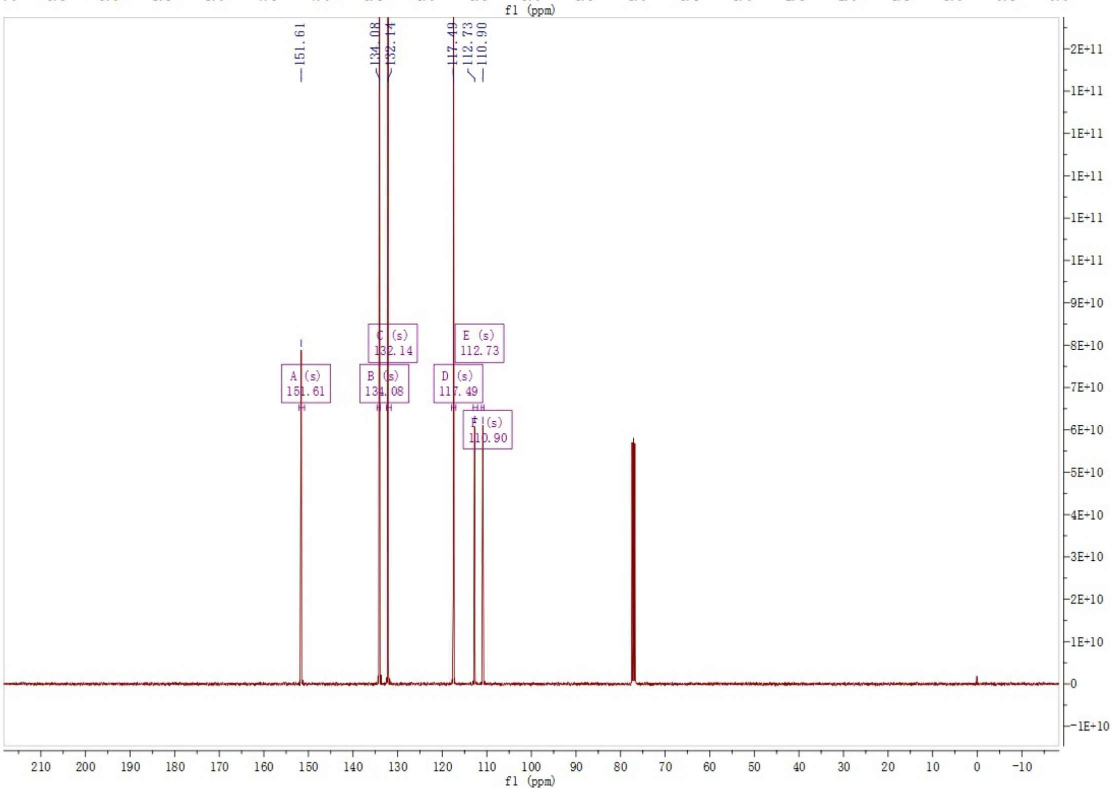

7a 2-bromo-4-nitrophenol

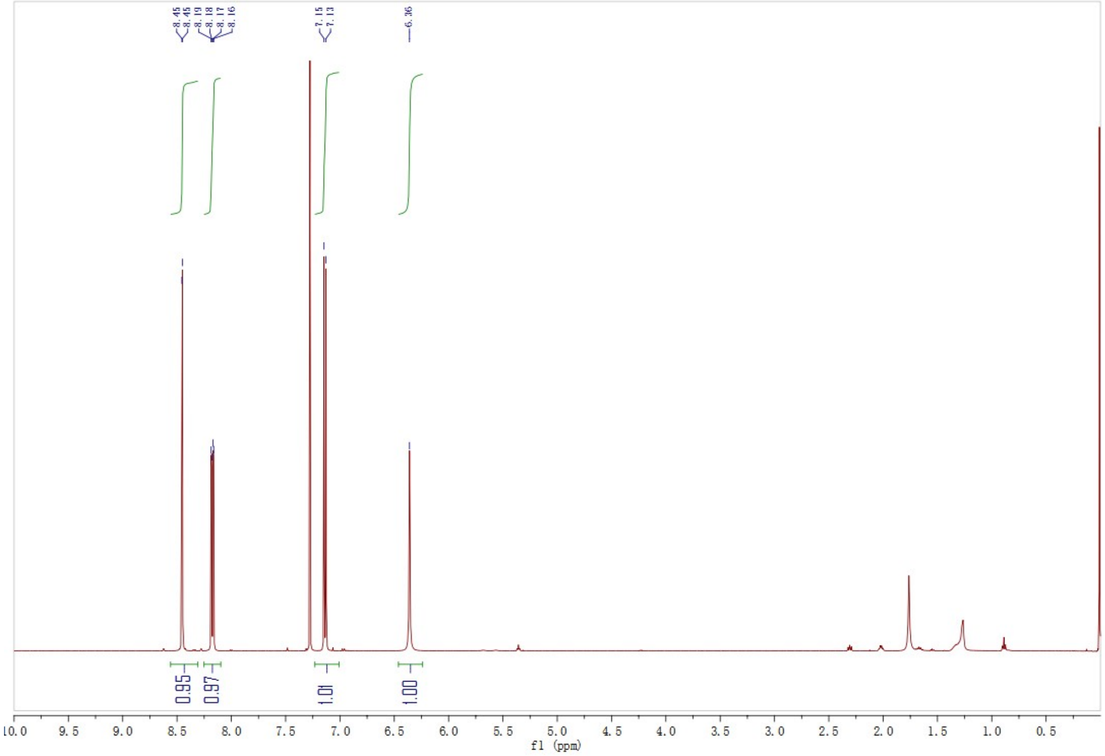

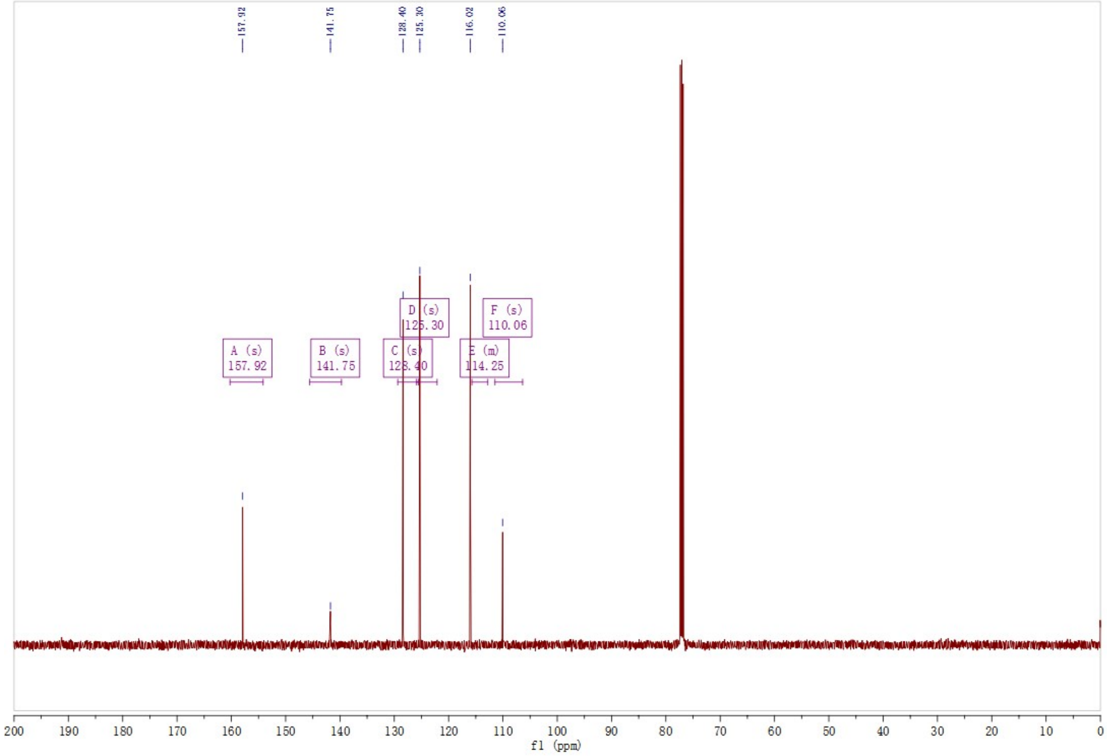

8a 4-bromo-2-methylphenol

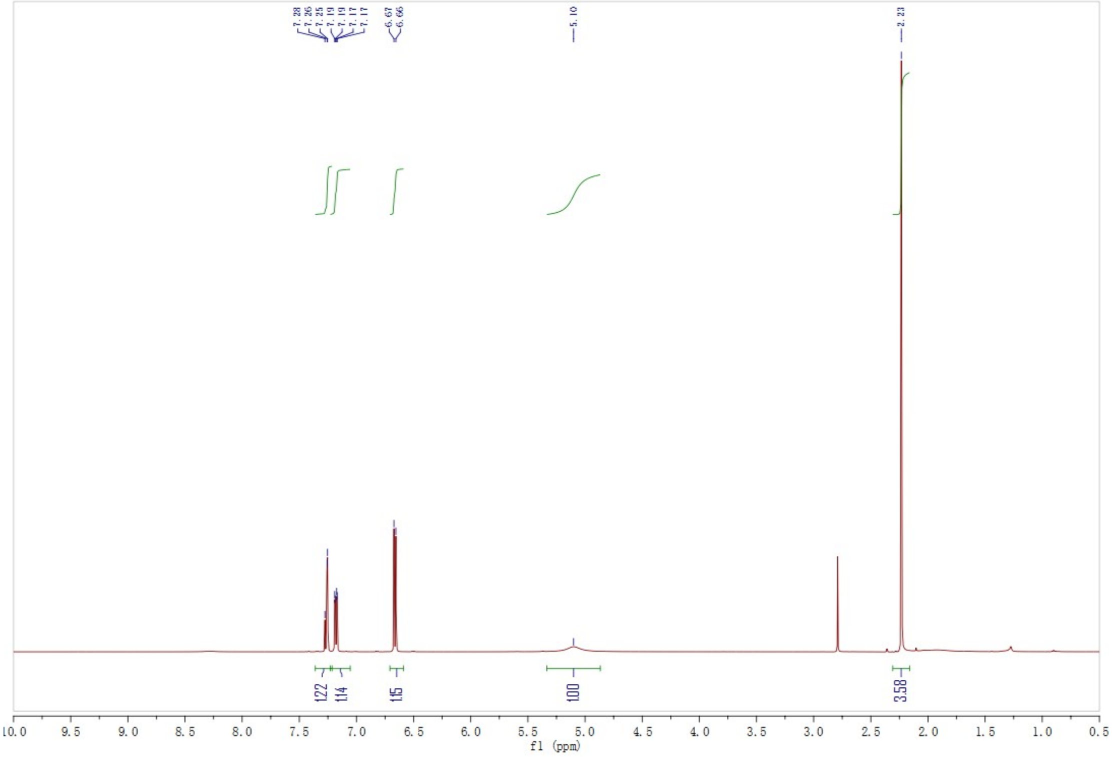

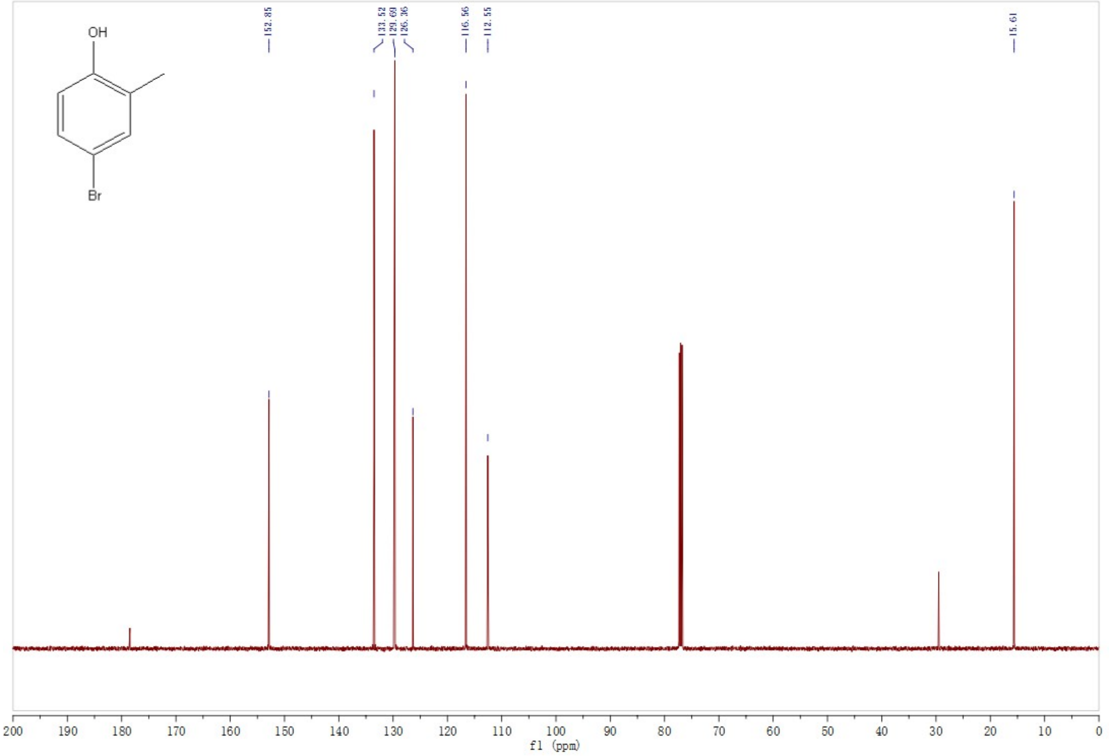

9a 4-bromo-2-(trifluoromethyl)phenol

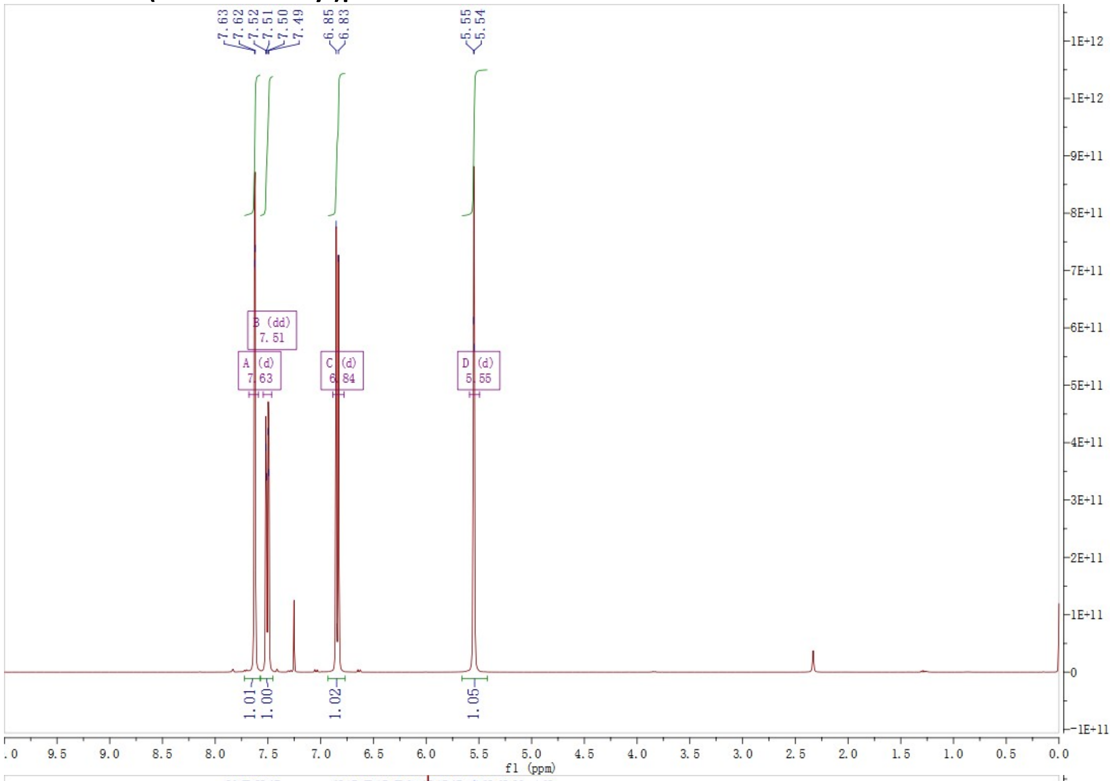

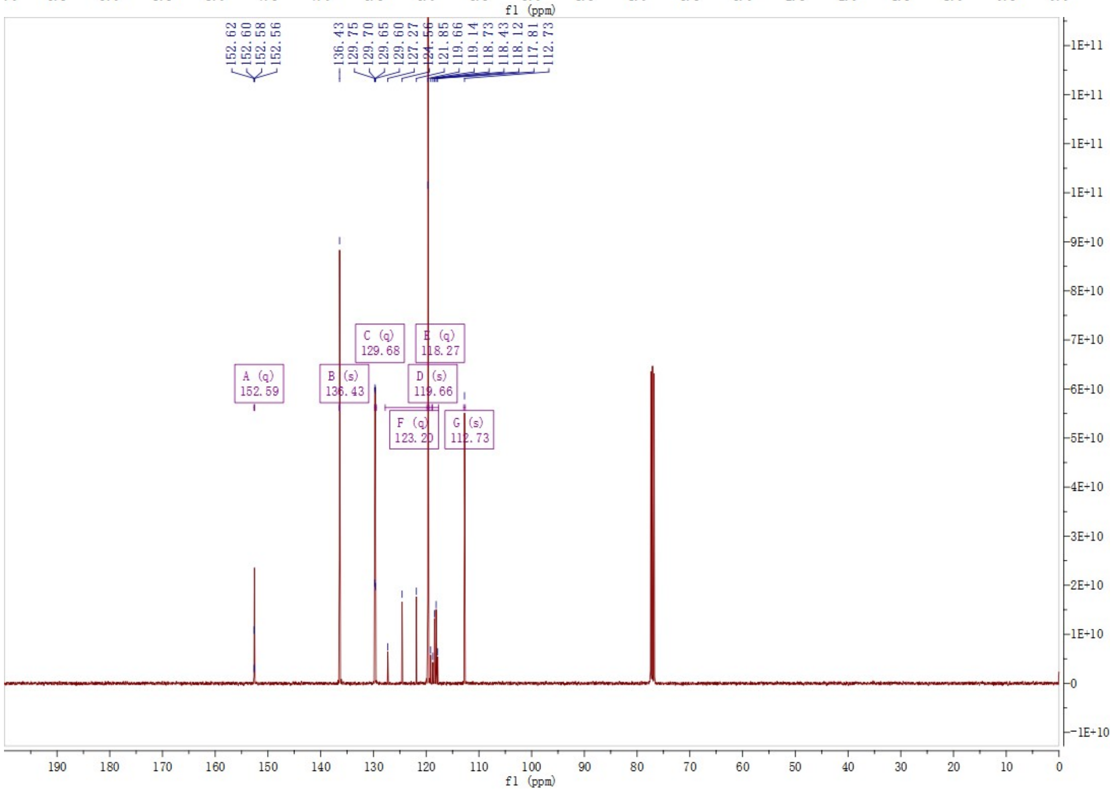

10a 4-bromo-2-fluorophenol

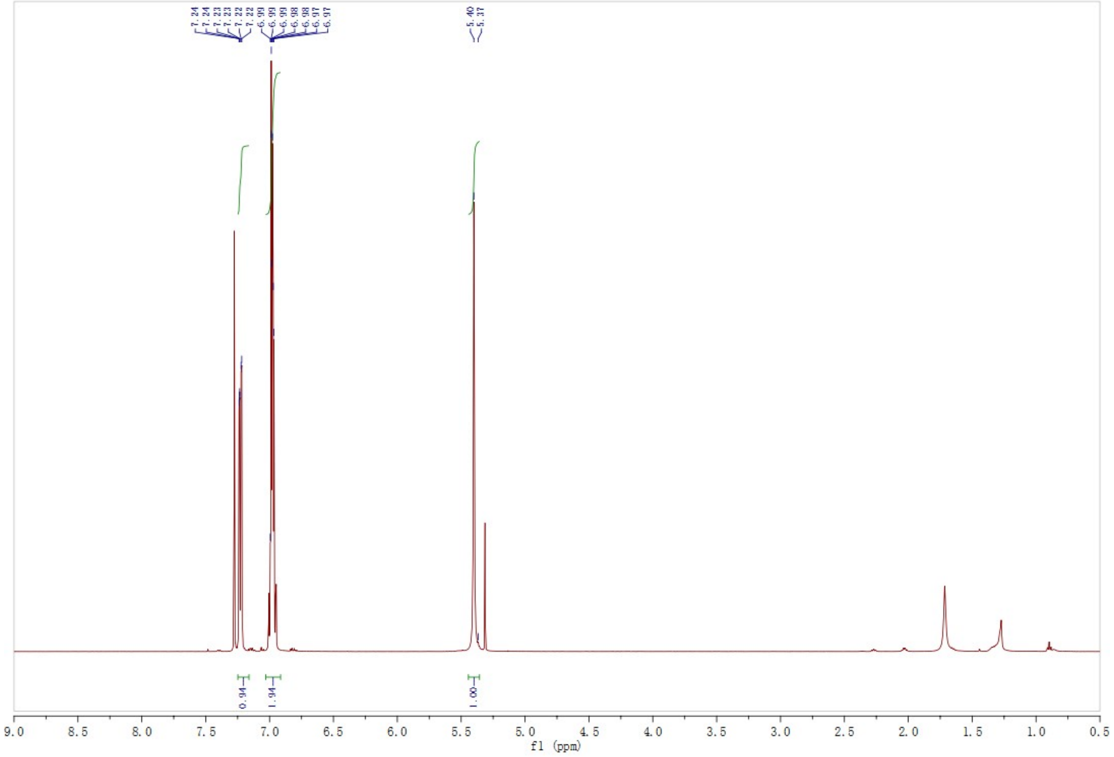

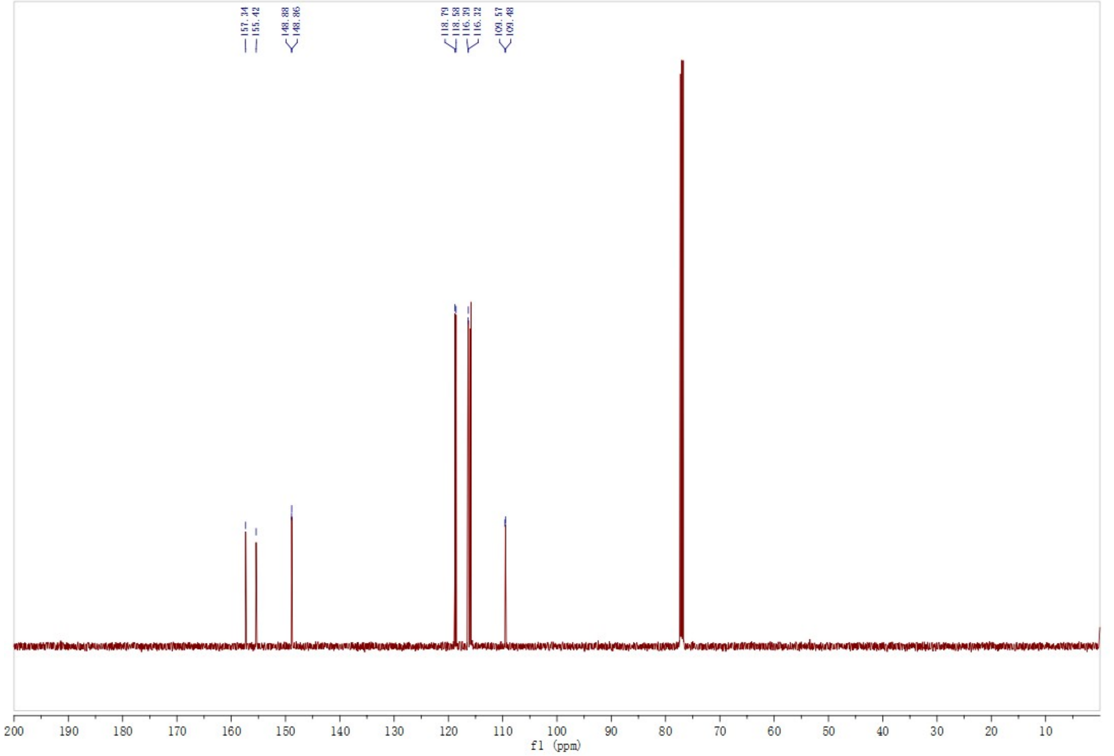

11a 4-bromo-2-chlorophenol

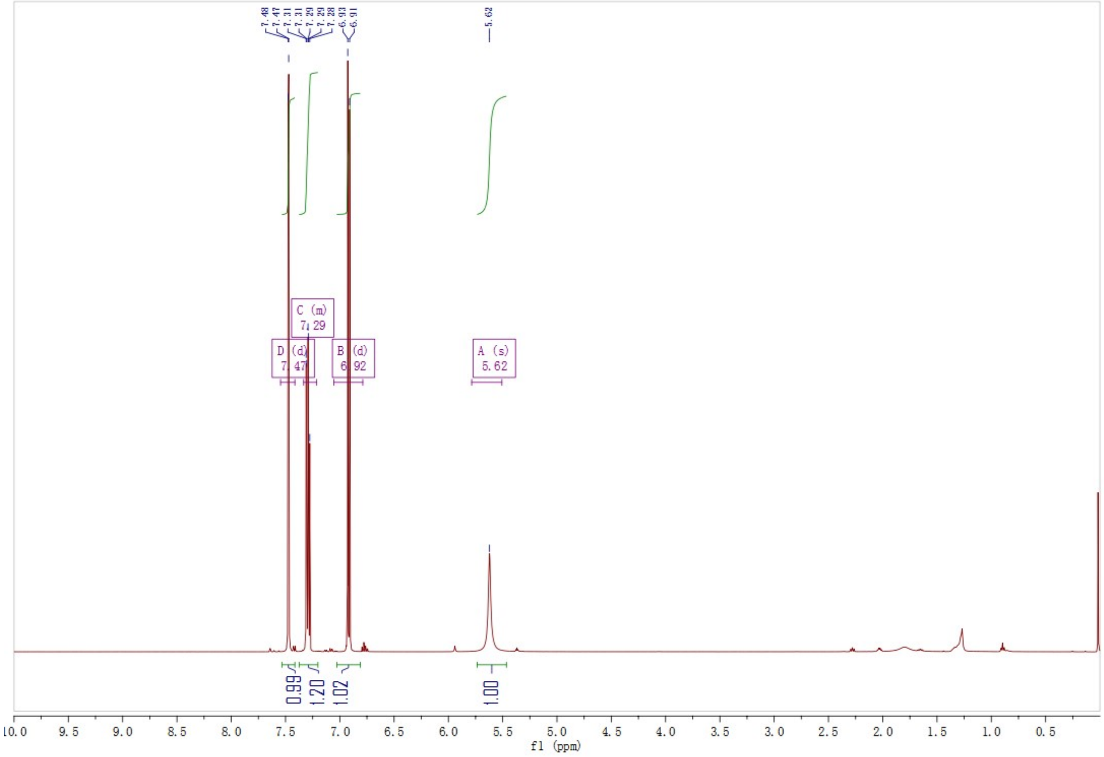

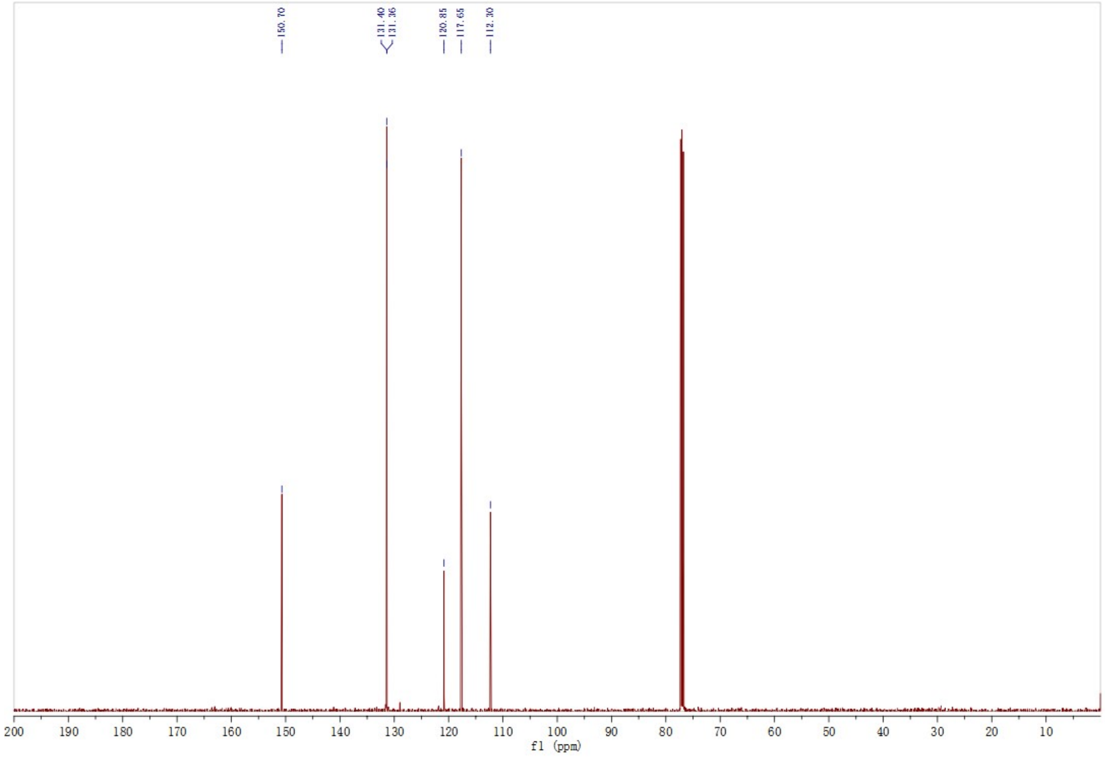

12a 2,4-dibromophenol

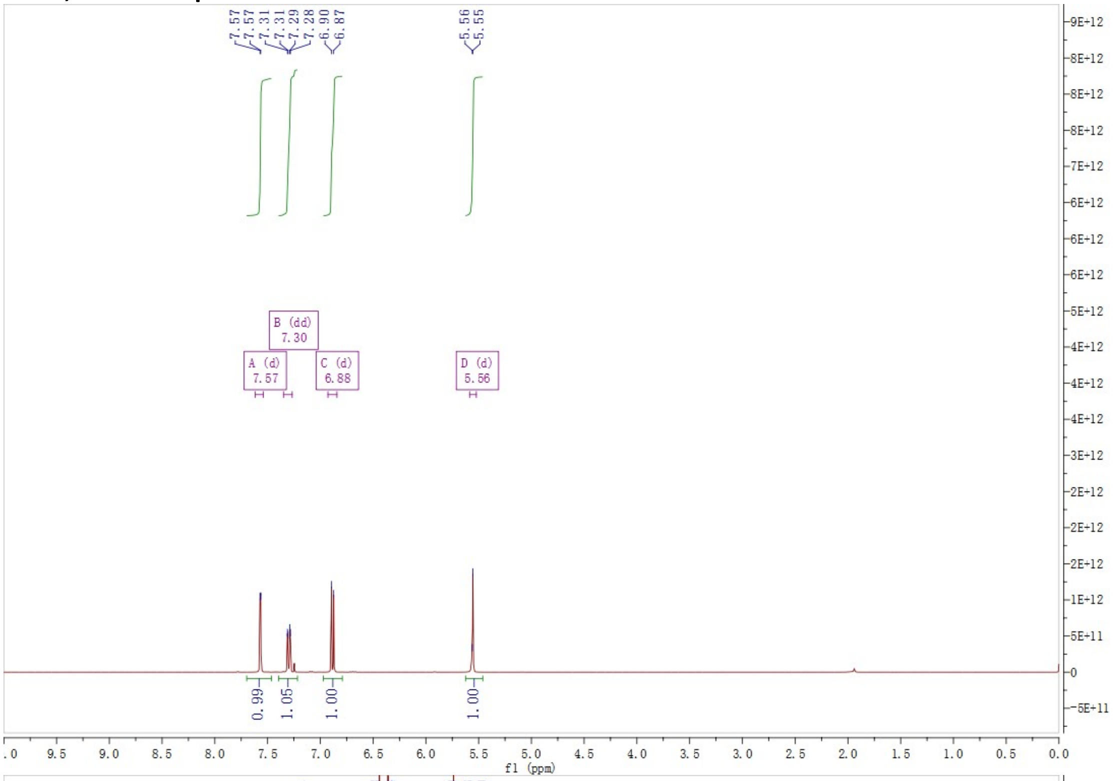

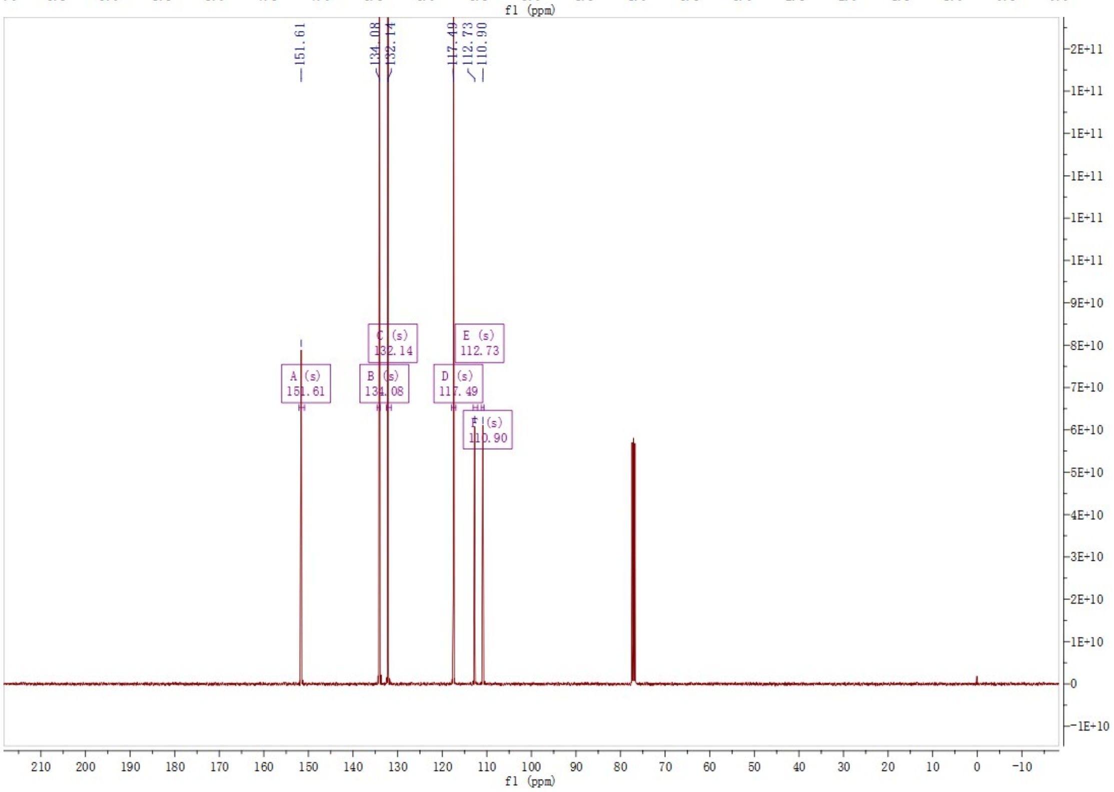

13a 4-bromo-2-iodophenol

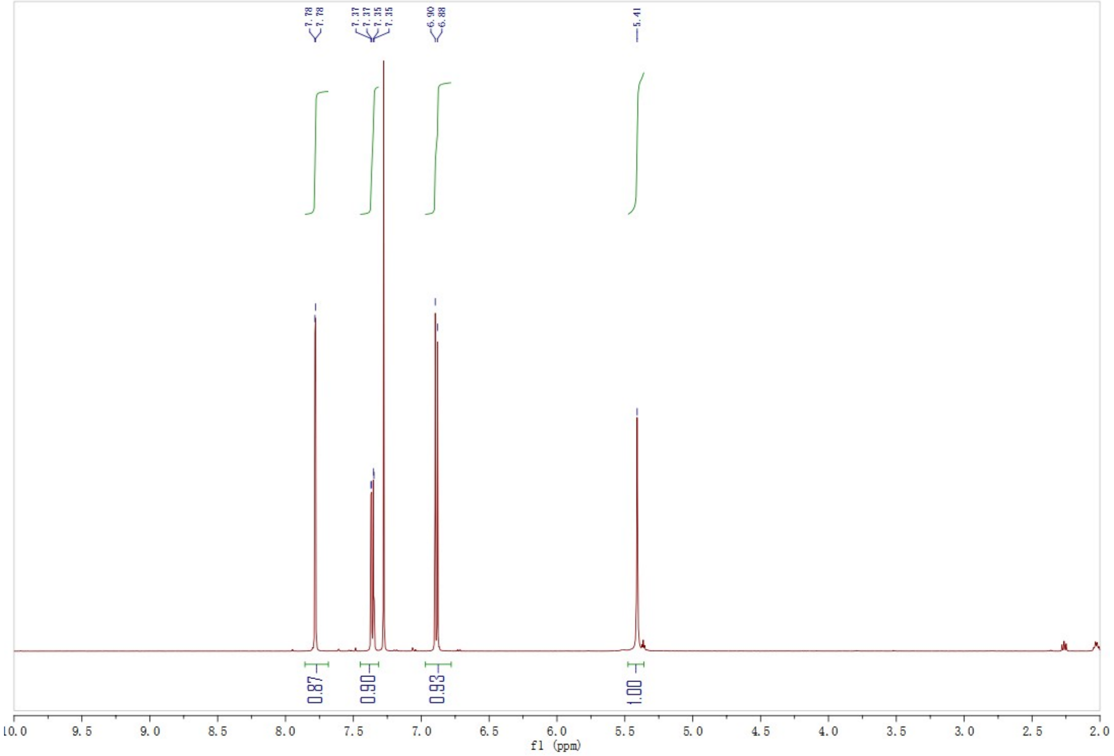

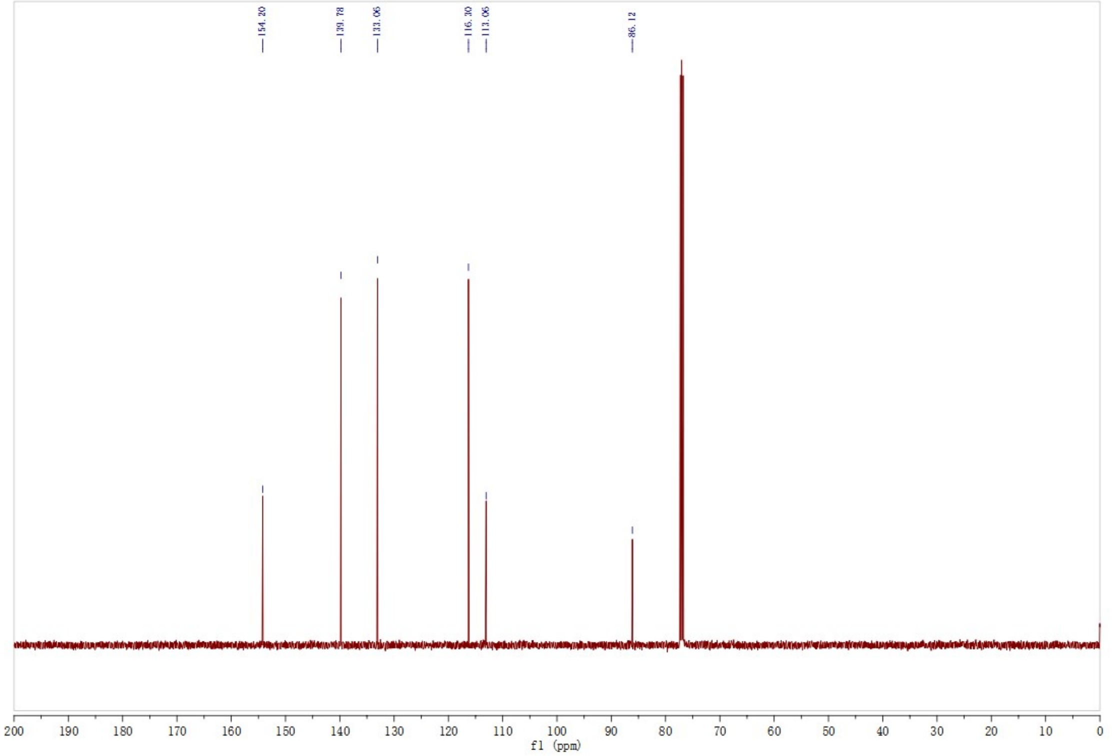

14a 4-bromo-3-methylphenol

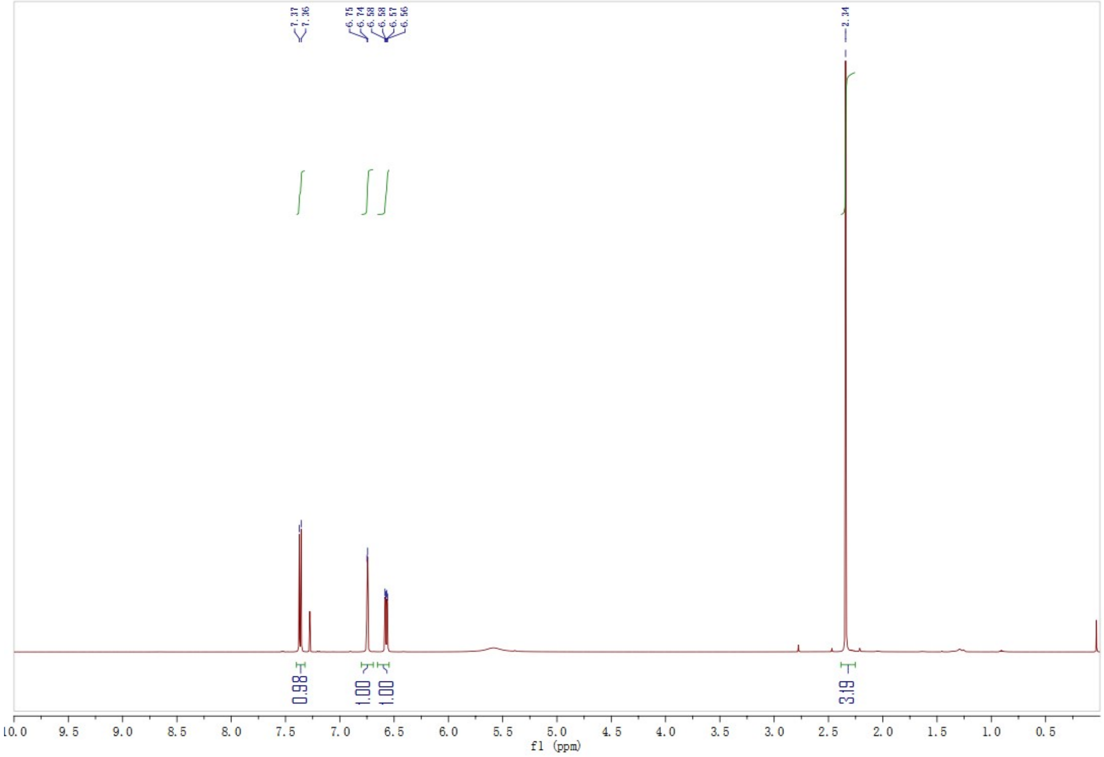

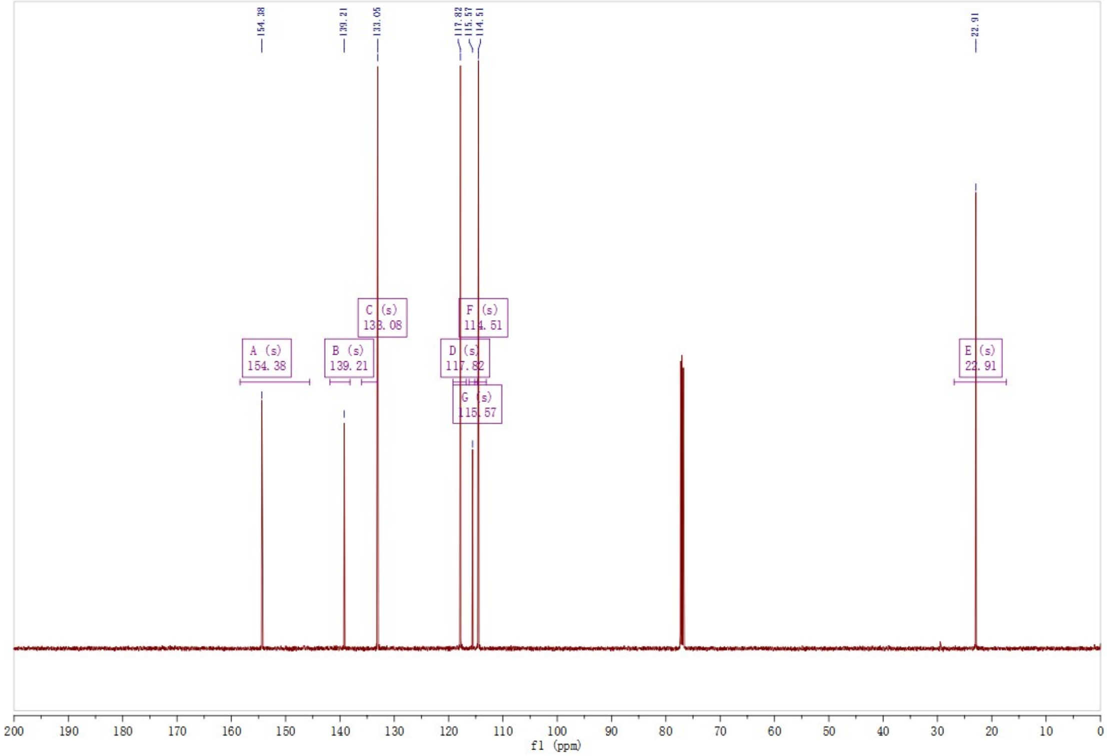

15a 4-bromo-3-(trifluoromethyl)phenol

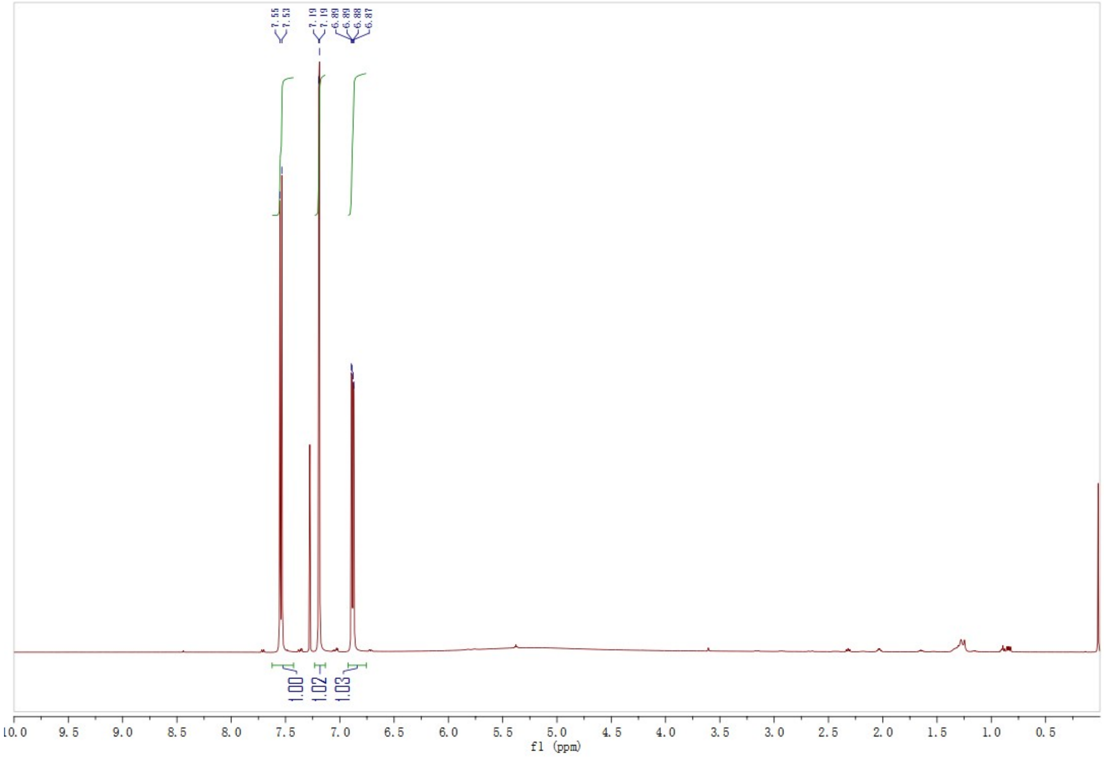

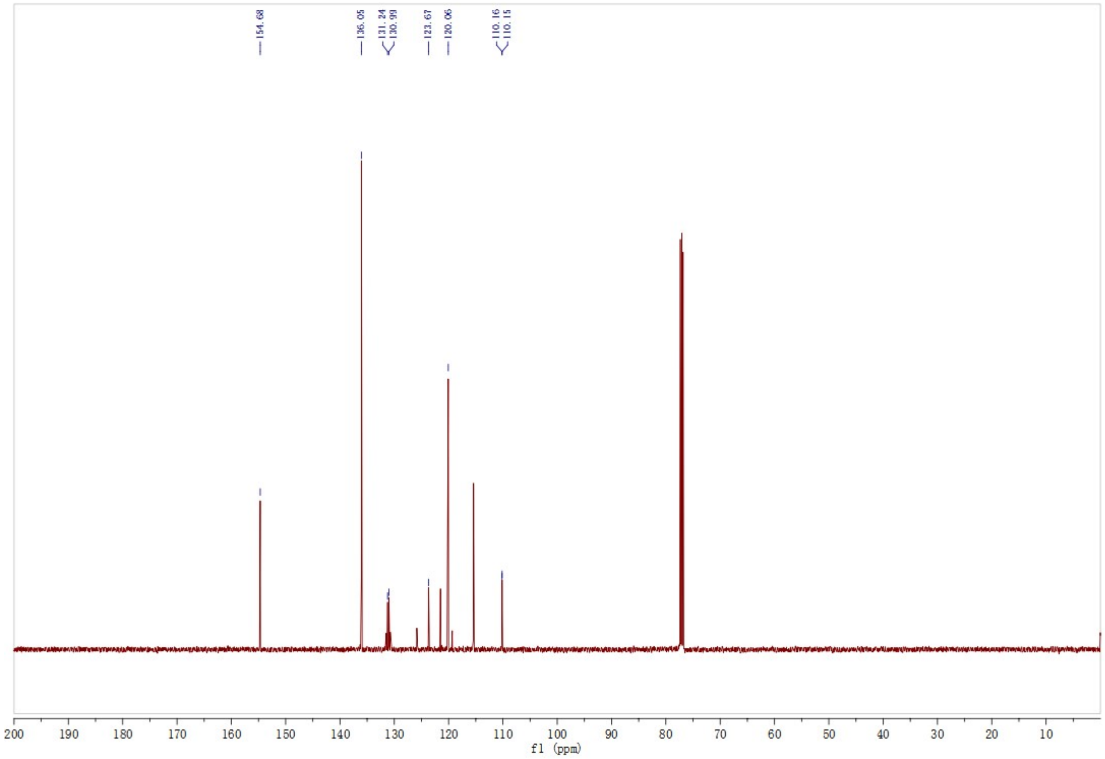

16a 4-bromo-3-fluorophenol

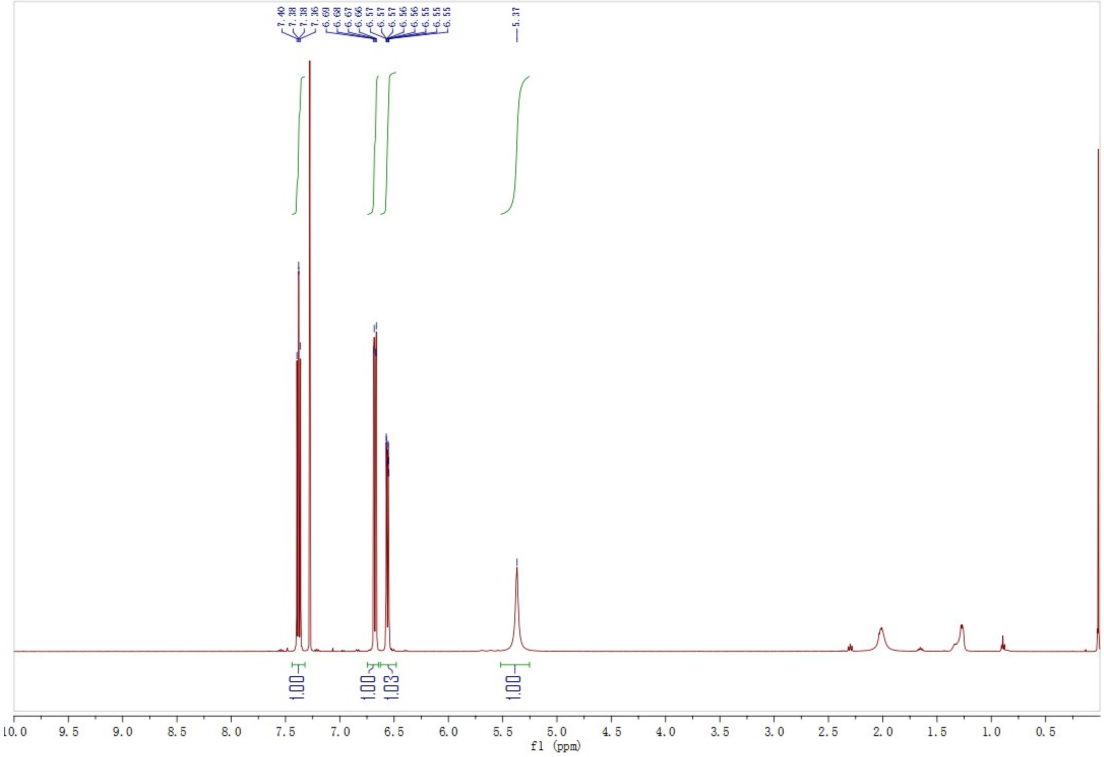

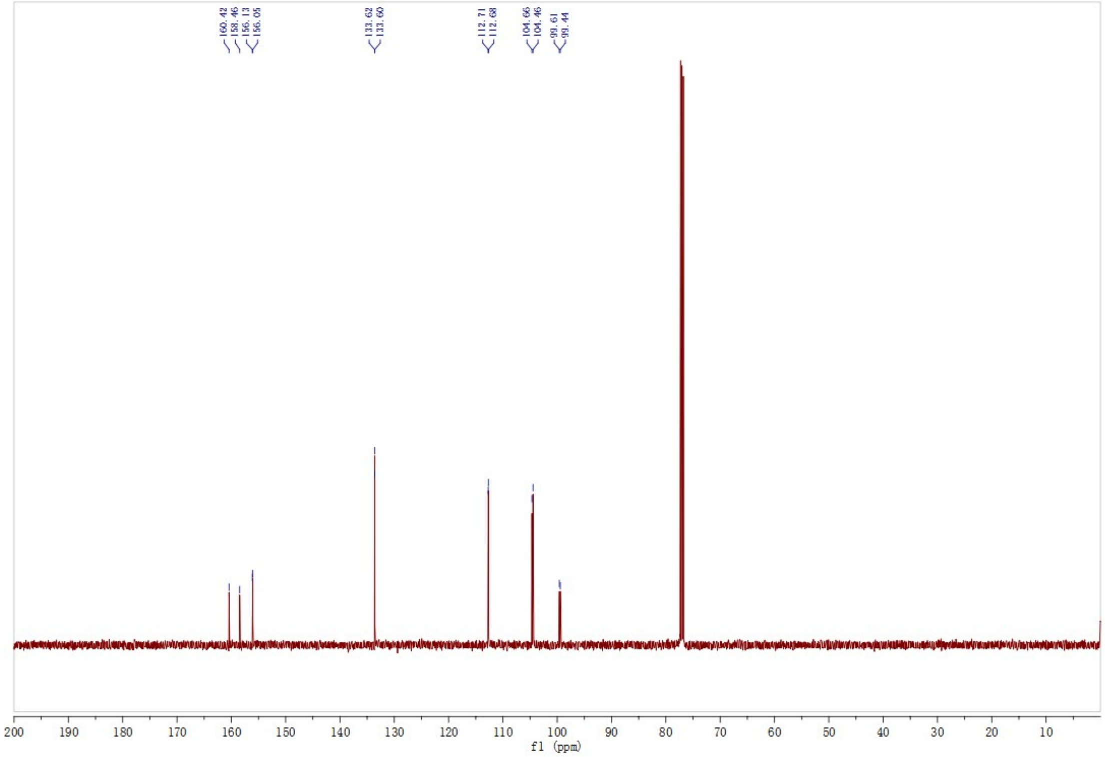

17a 4-bromo-3-chlorophenol

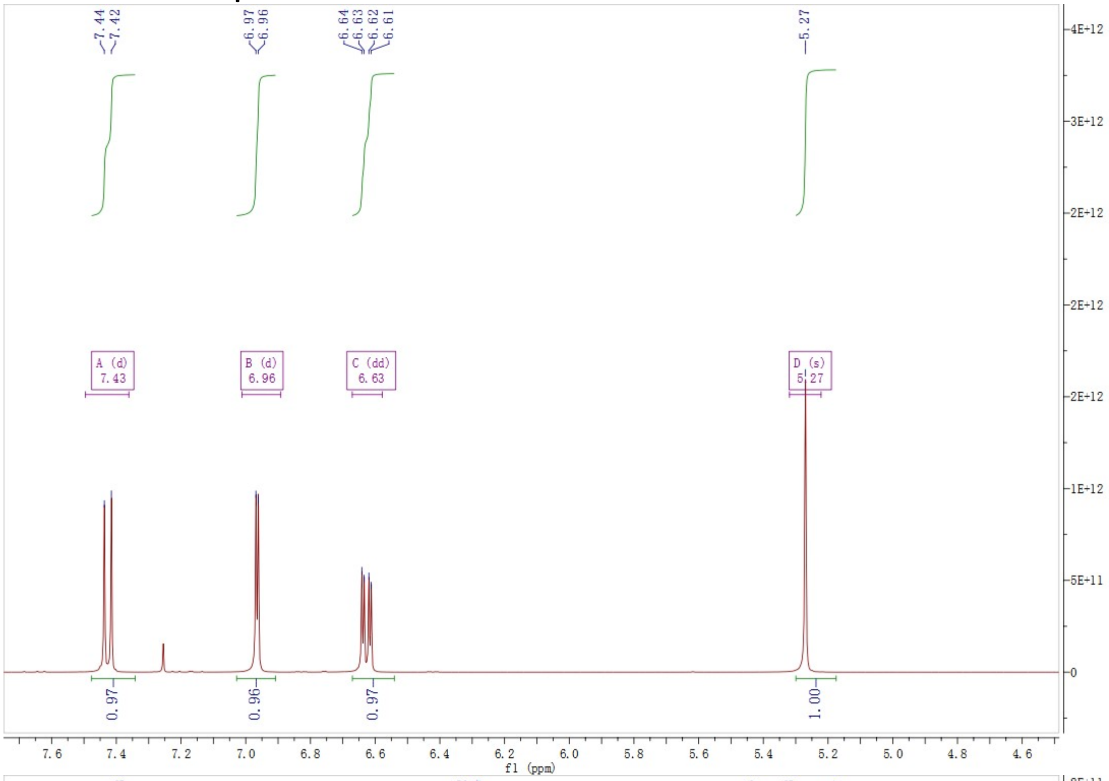

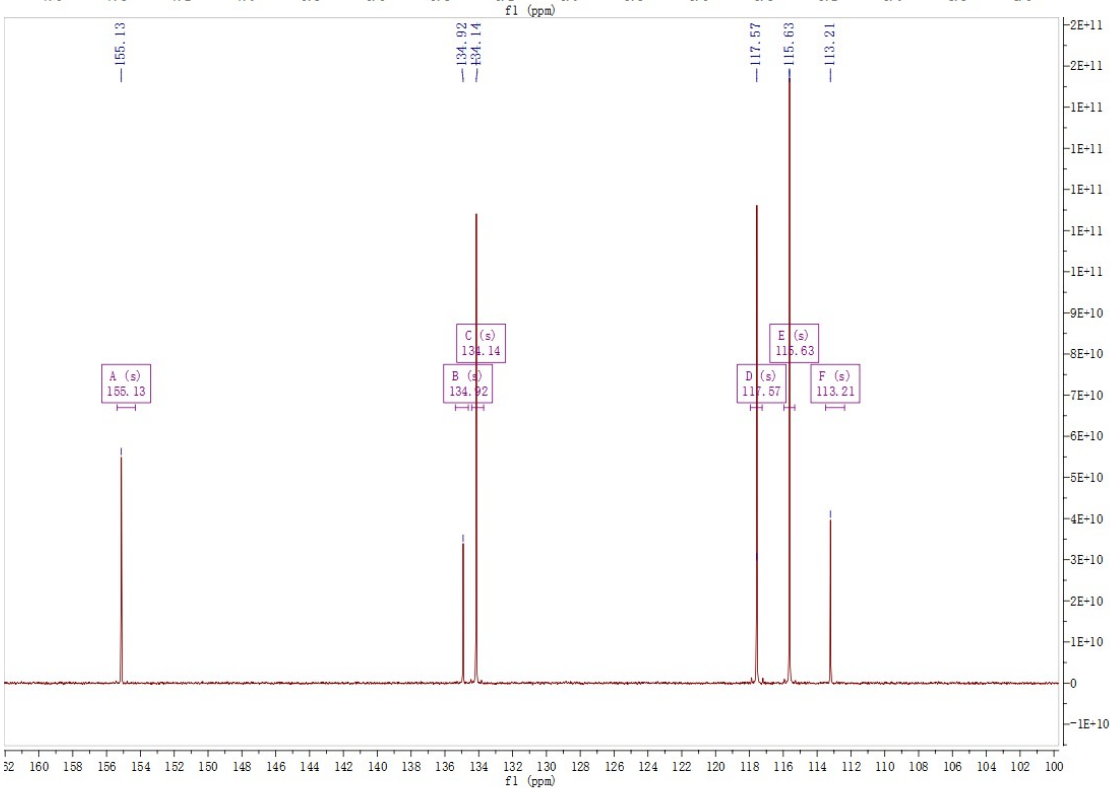

18a 4-bromo-2,6-dimethylphenol

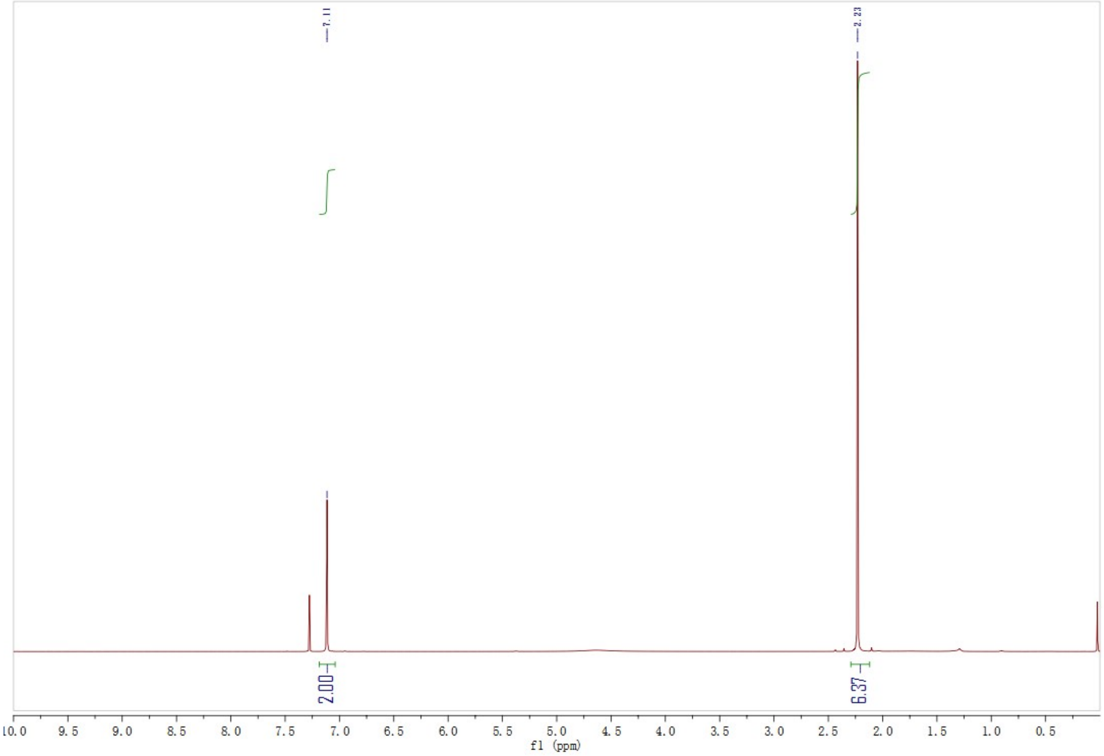

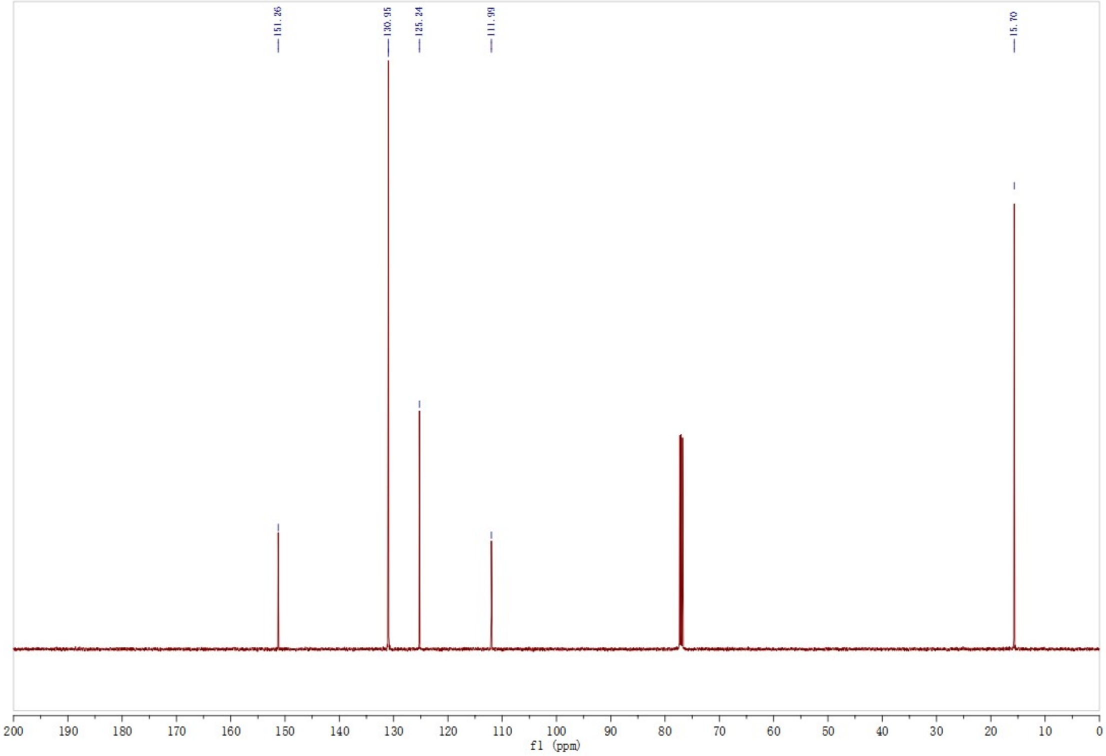

19a 4-bromo-3,5-dimethylphenol

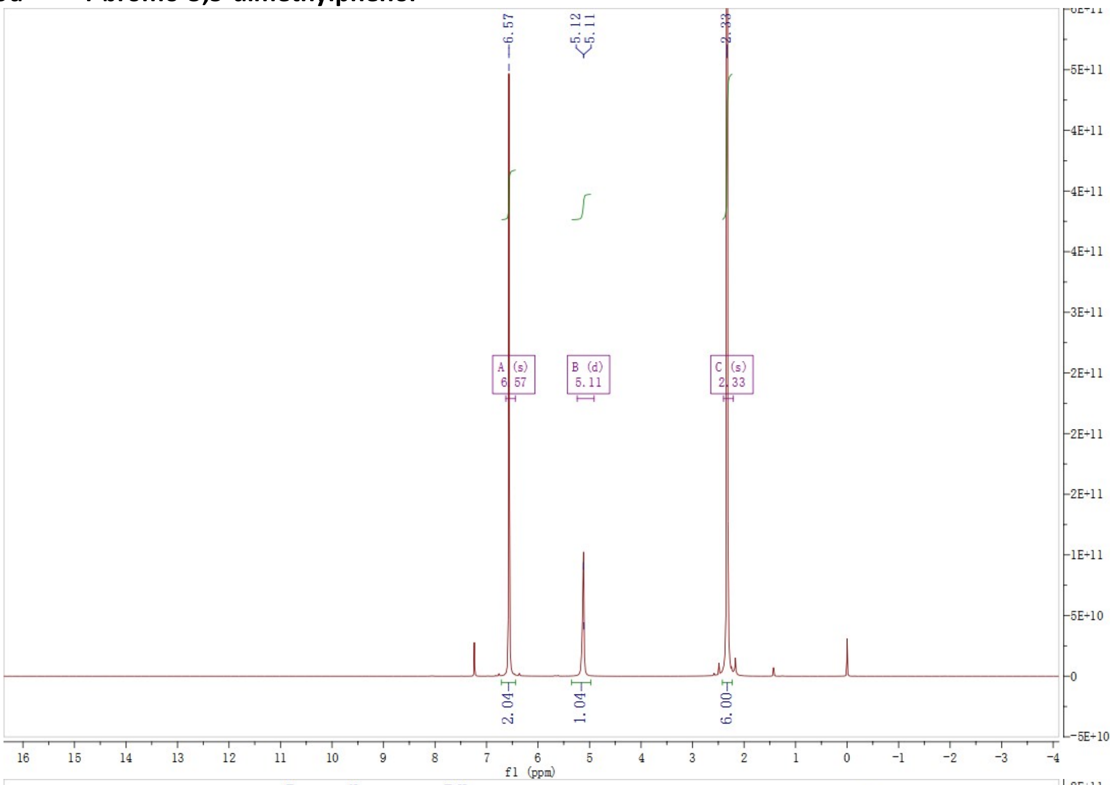

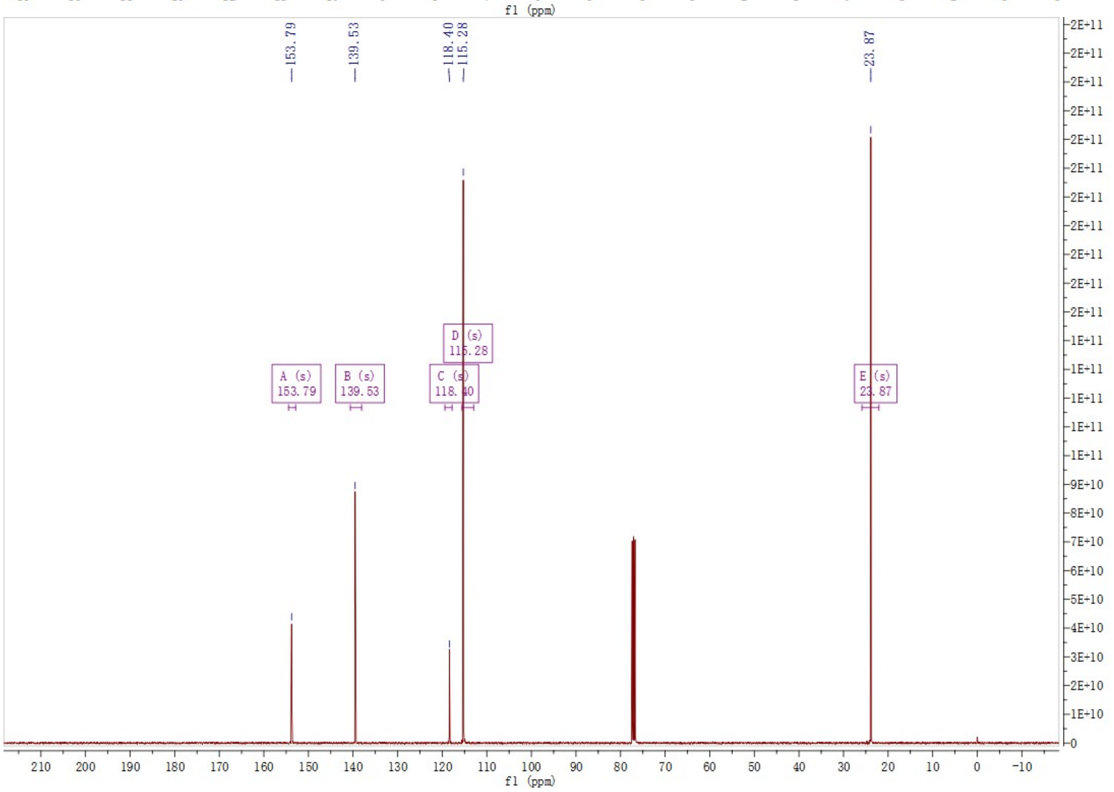

20a 4-bromo-2,6-difluorophenol

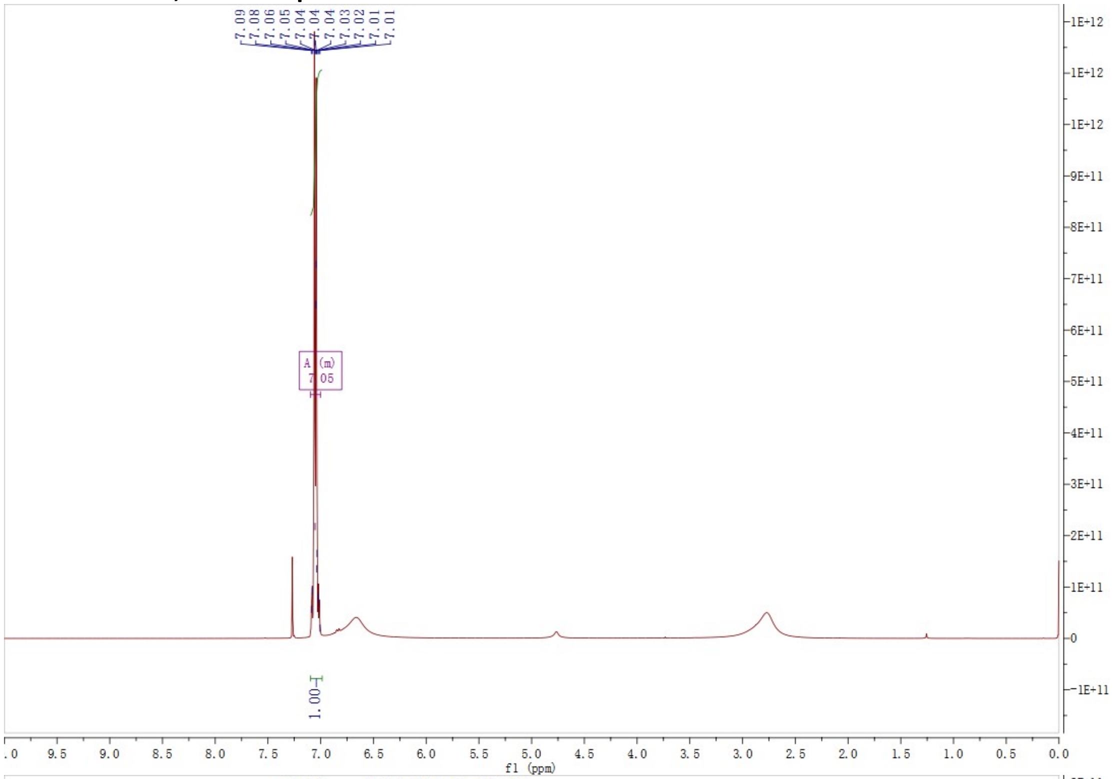

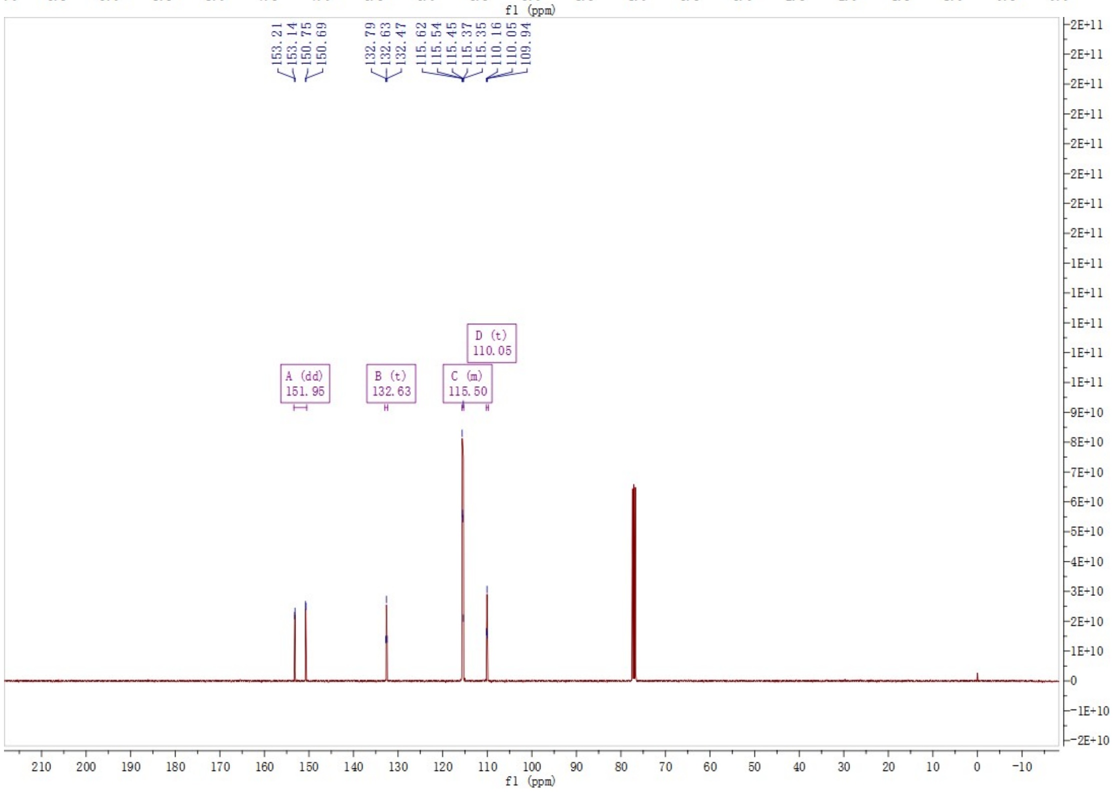

21a 4-bromo-3,5-difluorophenol

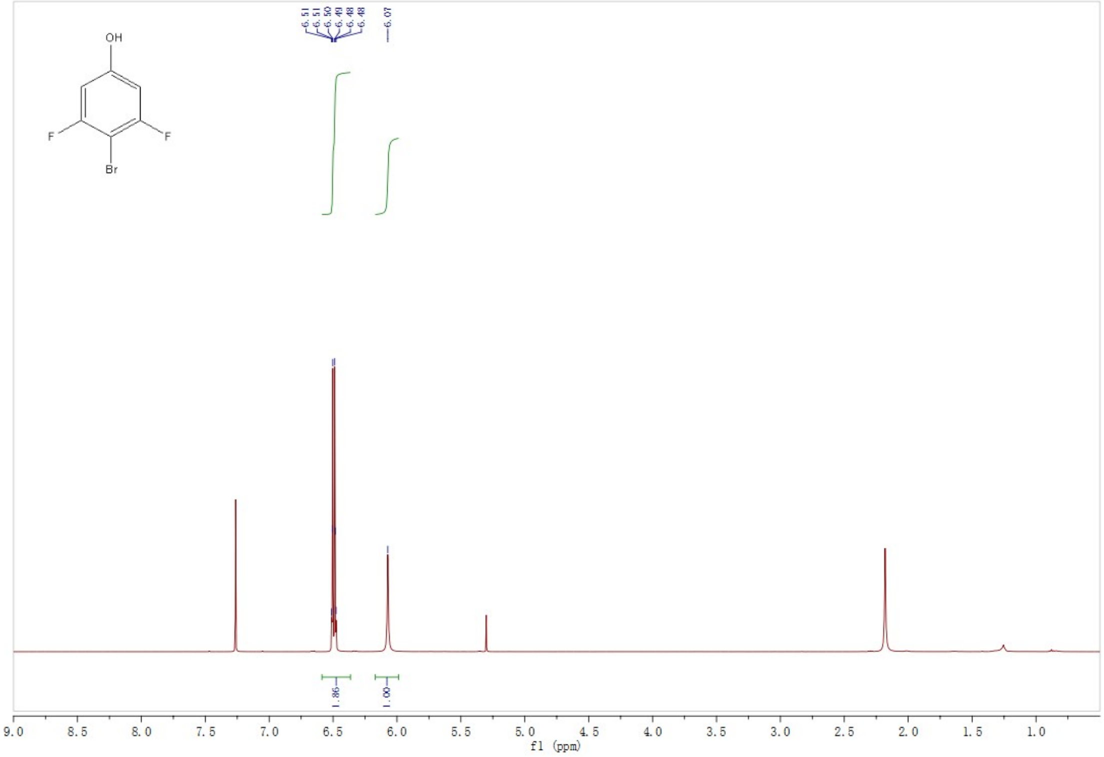

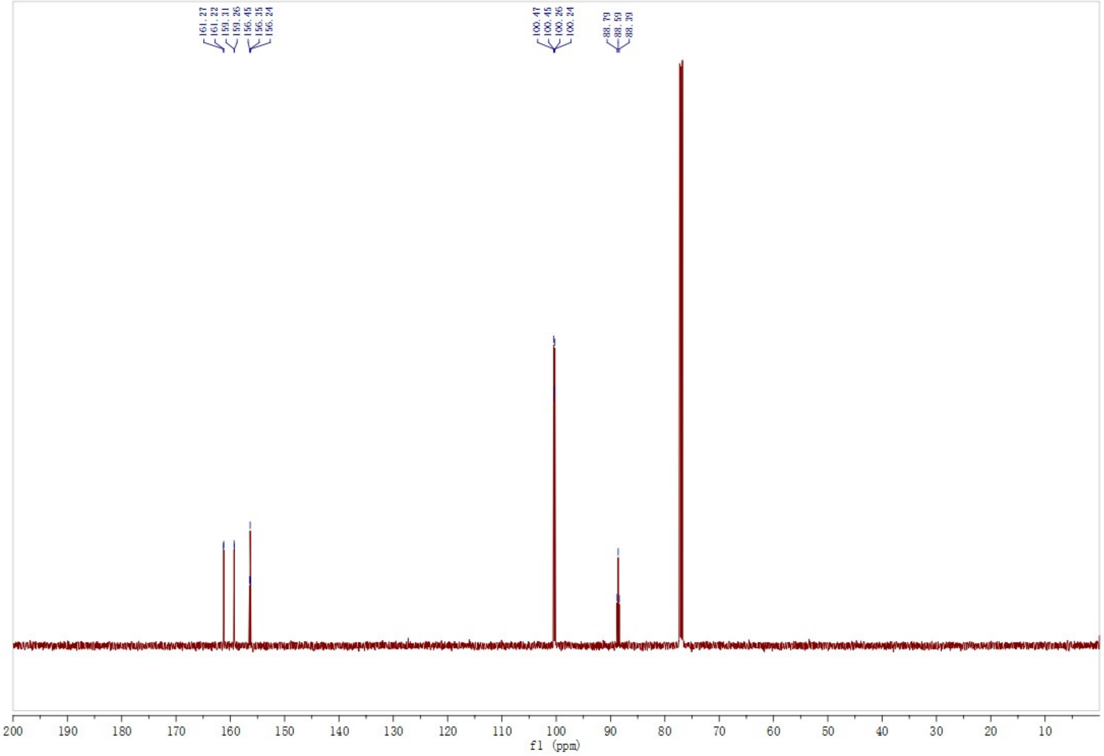

22a 4-bromo-2,3-difluorophenol

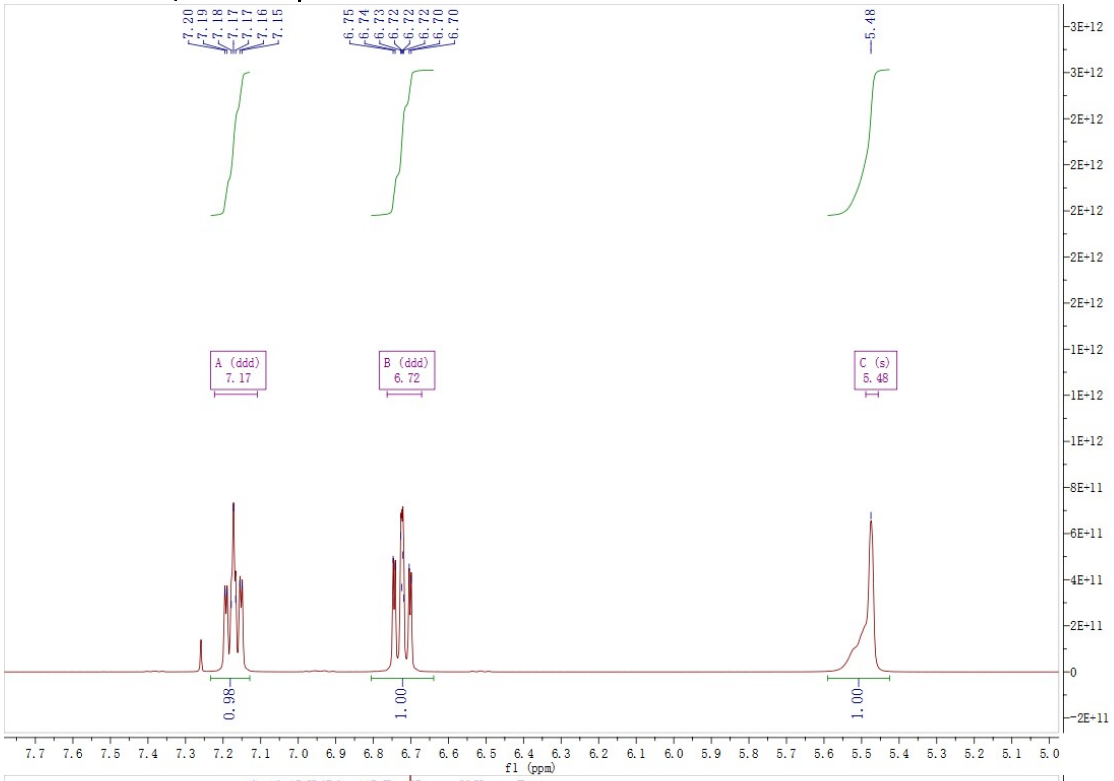

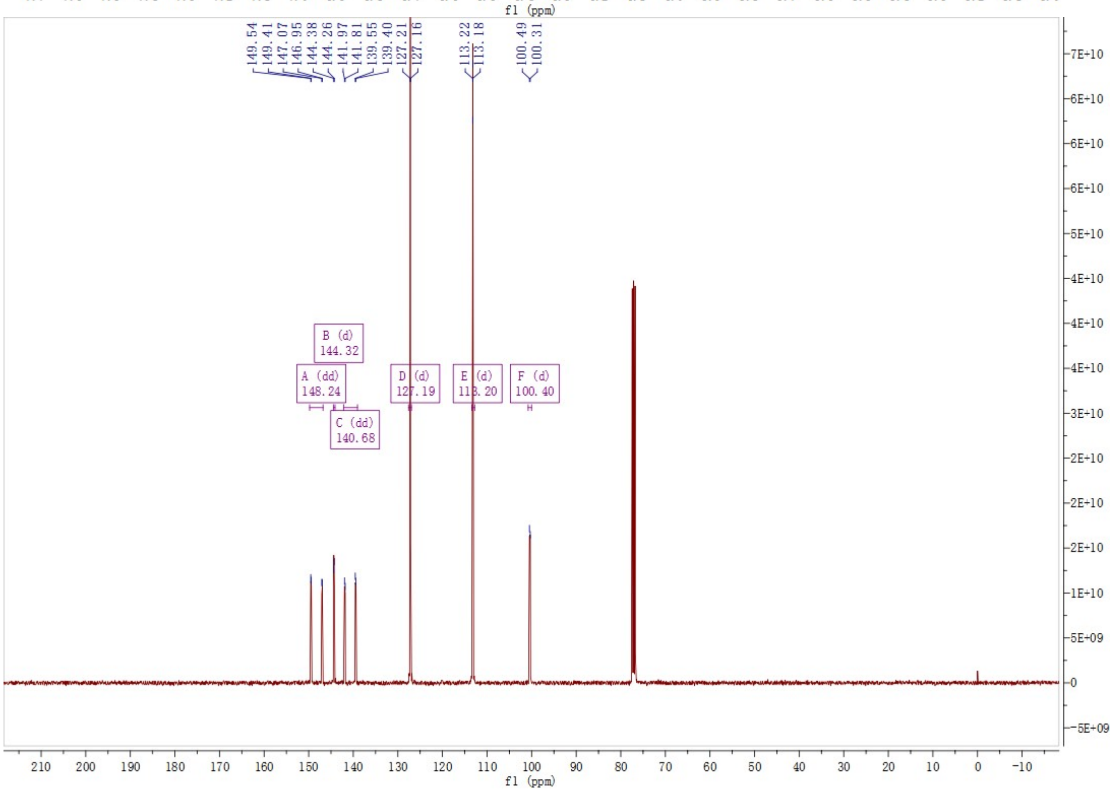

23a 1-bromonaphthalen-2-ol

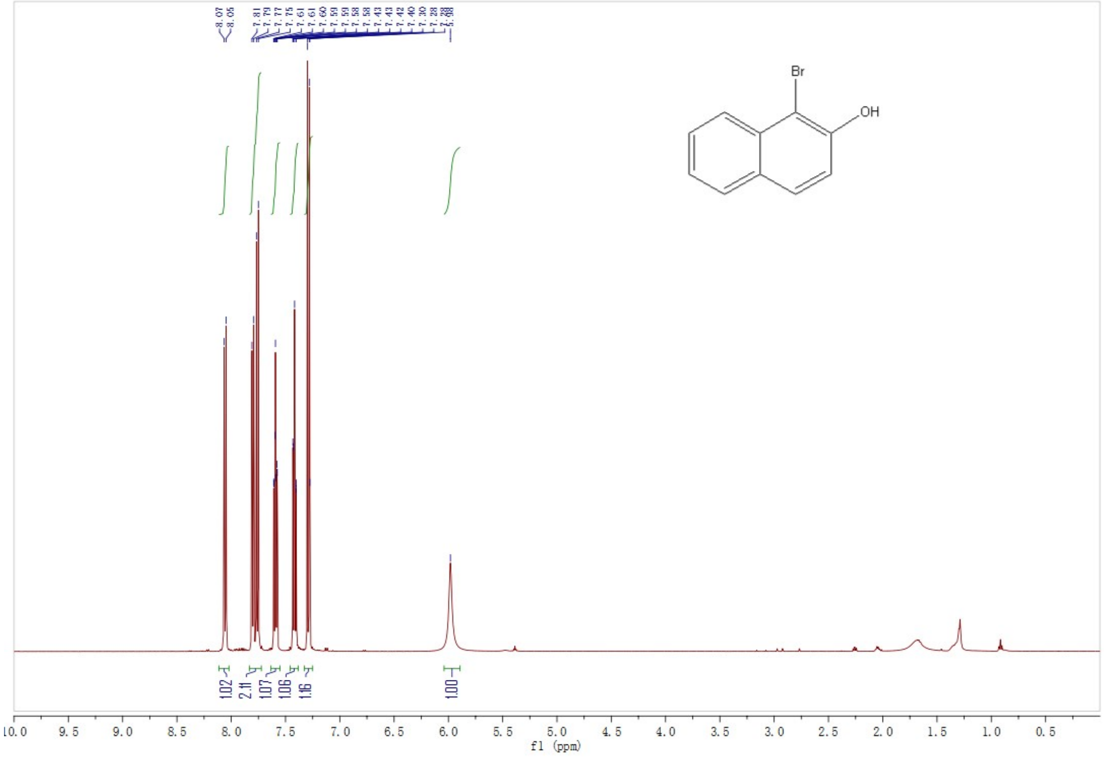

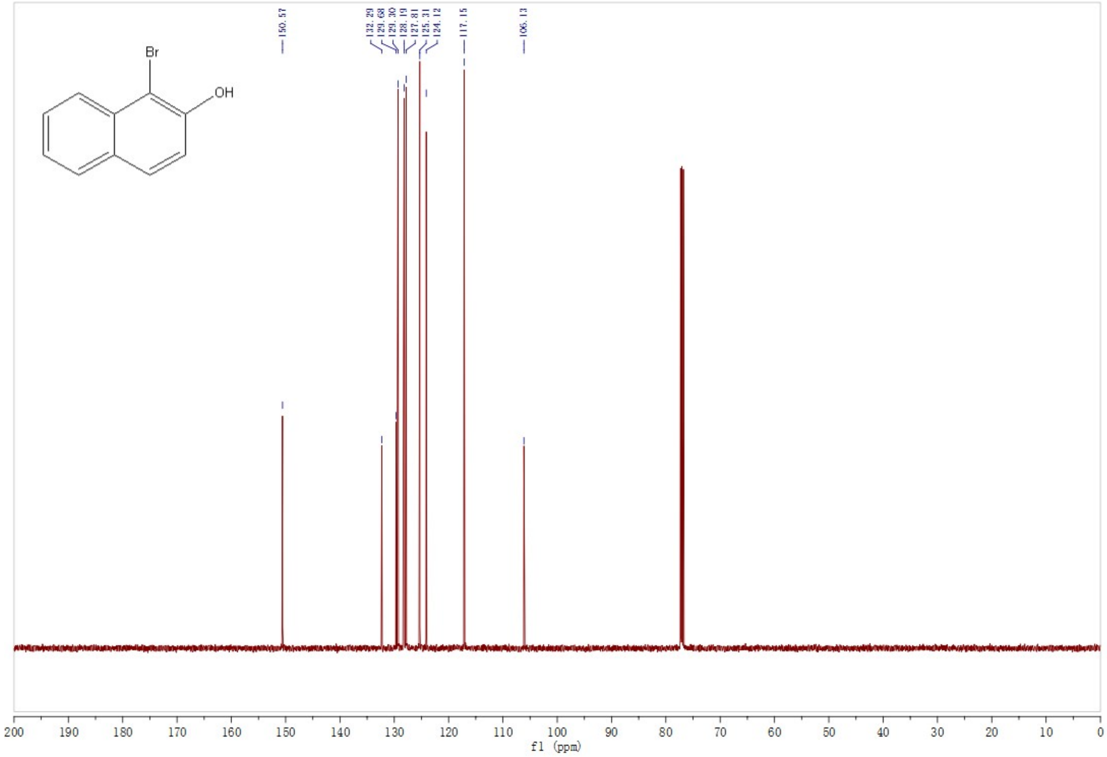

Supplement: Supplementary file 1 [file molecules-25-00914-s001.pdf]
